# Welfare states as lifecycle redistribution machines: Decomposing the roles of age and socio-economic status shows that European tax-and-benefit systems primarily redistribute across age groups

**DOI:** 10.1371/journal.pone.0255760

**Published:** 2021-08-25

**Authors:** Pieter Vanhuysse, Marton Medgyesi, Robert I. Gal

**Affiliations:** 1 Department of Political Science and Public Management, Danish Centre for Welfare Studies (DaWS), University of Southern Denmark, Odense, Denmark; 2 Danish Institute for Advanced Study (DIAS), University of Southern Denmark, Odense, Denmark; 3 Interdisciplinary Centre on Population Dynamics (CPop), University of Southern Denmark, Odense, Denmark; 4 Corvinus Institute for Advanced Studies, Corvinus University, Budapest, Hungary; 5 Child Opportunities Research Group, Centre for Social Sciences, Budapest, Hungary; 6 Hungarian Demographic Research Institute, Budapest, Hungary; FAME|GRAPE, POLAND

## Abstract

Social scientists identify two core functions of modern welfare states as redistribution across (a) socio-economic status groups (Robin Hood) and (b) ‘the lifecycle’ (the piggy bank). But what is the relative importance of these functions? The answer has been elusive, as the piggy bank is metaphorical. The intra-personal time-travel of resources it implies is based on non-*quid-pro-quo* transfers. In practice, ‘lifecycle redistribution’ must operate through inter-age-group resource reallocation in cross-section. Since at any time different birth cohorts live together, ‘resource-productive’ working-aged people are taxed to finance consumption of ‘resource-dependent’ younger and older people. In a novel decomposition analysis, we study the *joint* distribution of socio-economic status, age, and respectively (a) all cash and in-kind transfers (‘benefits’), (b) financing contributions (‘taxes’), and (c) resulting ‘net benefits,’ on a sample of over 400,000 Europeans from 22 EU countries. European welfare states, often maligned as ineffective Robin Hood vehicles riddled with Matthew effects, are better characterized as inter-age redistribution machines performing a more important second task rather well: lifecycle consumption smoothing. Social policies serve multiple goals in Europe, but empirically they are neither primarily nor solely responsible for poverty relief and inequality reduction.

## Introduction: The welfare state as Robin Hood and piggy bank, beyond metaphor

Welfare states have evolved since their inception in late nineteenth-century Europe into sizable and resource-consuming institutions. Today, total social spending in Europe takes up around 28 percent of GDP on average and around 66 percent of total government revenue, and it affects many aspects, and every stage, of citizens’ lives. But what do welfare states mostly do? Two social science approaches have been dominant in answering this question. Standard economic accounts view welfare states’ primary role as resolving market failures, not least because of the state’s unique ability to avoid moral hazard and adverse selection by pooling risks widely and making participation obligatory [1–4]. Barr [1] pointed out that alongside the welfare state’s familiar redistributive function is its efficiency role in areas where, because of market failures, private markets would produce inefficiently or not at all.

Markets fail whenever property rights are incomplete (imperfect excludability or non-transferability), when information and transaction costs are too high, or when the parties are unable to agree upon the terms of exchange due to structural bargaining problems. As a result, private actors cannot enter mutually fruitful transactions such as insurance against the risk of unemployment, disability, and health deterioration because of asymmetric information, but also moral hazard (unemployment and medical insurance) and probabilities too close to 1 to insure (medical insurance); they cannot attain optimal levels of expenditures on education (because of externalities on the outcomes, but also imperfect capital markets); and they cannot obtain insurance against the unpredictability of longevity (because of the uninsurability of related risks such as future inflation).

Standard sociological and public policy accounts, in turn, view the welfare state as a political Robin Hood of sorts; a redeemer of markets and families. Welfare states, in this view, are analyzed primarily as a tool for poverty relief, redistribution from higher socio-economic status (SES) groups to lower-SES groups, and inequality reduction. The classic question here has been to what degree various types of welfare states perform this task effectively (e.g., [5–7]; for a critical synthesis, see [8]). Strictly speaking, the efficiency function implies that a welfare state of some sort would be necessary even if all poverty could magically be removed (Barr [1]). This offers a re-interpretation of the finding that the beneficiaries of welfare state benefits include the middle class [5–7]. Others in the sociology and public policy tradition have added that welfare states, more generally, temper the social costs of market forces through wider social citizenship rights and reduce citizens’ material dependence on markets [9,10]. The welfare state, according to this view, ‘decommodifies’ people: it ‘maintain[s] a livelihood without reliance on the market’ and it does so ‘as a matter of right’ [10]. In a fundamental new focus on the household economy, Esping-Andersen [11–13] later emphasized how welfare states can also ‘defamilialize:’ they can render individuals, especially women, more independent from their family.

After their initial formulations, both approaches have incorporated a lifecycle perspective. The start of the social investment paradigm from the late 1990s onward has refocused the attention of sociologists and policy researchers on how, early on in the lifecycle, ‘predistributive’ social policies such as education, lifelong learning, and activation can boost individuals’ ability to adjust to changing market demands and to earn market incomes, thereby preventing many social problems (‘preparing’) rather than dealing with them (‘repairing’) later [11,12,14] but see [15,16]. Birnbaum et al. [17] measure how social policies, seen as ‘generational welfare contracts,’ balance program replacement rates across different lifecycle stages. And in economics, while Barr [1] identified the separate rich-to-poor redistributive and efficiency functions of the welfare state, Barr [18] famously added a further key purpose: redistribution ‘over the lifecycle.’ For the rich-to-poor and lifecycle redistribution functions, Barr [18] introduced respectively the ‘Robin Hood’ and ‘piggy bank’ terms. The piggy bank is made necessary by a fundamental, one might say universal, lifecycle consumption financing problem, which members of each successive generation need to solve. There is a discrepancy in the typical paths of labor income and consumption [19,20]. Productivity and earning powers are heavily concentrated in the middle of the lifecycle—a hump-shaped curve—but people have to consume in childhood and in old age, too, when they do not earn much primary income. According to the piggy bank interpretation, a significant part of the welfare state consists of enabling individuals to make transfers between ‘their own selves’ at different stages of their lives.

But which of these two core functions is *more* important—Robin Hood or piggy bank? Elementary though this question might seem for any proper understanding of how welfare states operate, there is no straightforward research design to answer it. The reason is that the piggy bank is largely metaphorical. The ‘lifecycle redistribution’ it entails is a legally and methodologically elusive concept. The time-travel of resources implied by the piggy bank’s ‘lifecycle redistribution’ is not a well-defined system of detectable, *quid-pro-quo* exchanges imparting reliable and legally enforceable property rights connecting the same person over time. Two concepts of ‘lifecycle redistribution’ are invoked here: redistribution between first, different stages of one’s own life (one’s successive selves) and second, groups of different ages at a given moment (age groups in cross-section) (e.g. [21–23]. The first concept can be seen as the individuals’ objective, the second as the mechanism by which the welfare state assists the achievement of that objective [24]. As Samuelson [25] noted, in reality, no direct intertemporal *intra*-personal links (the first concept) can be established. Simply put, short of Robinson Crusoe solutions (storing current production for one’s own future consumption by stockpiling bricks or non-perishable goods, say, tuna cans), there cannot be any intertemporal reallocations between one single person’s selves over his/her lifetime *without* making inter-age group transactions. The only alternative is cross-sectional: to exchange one’s current production for a claim on future production by younger generations (the second concept). This can be done either by saving to accumulate assets to be sold later to younger generations, or by obtaining a promise of a share of future production [18,24,26].

To illustrate this, consider a classic example of ostensible piggy bank redistribution ‘over the lifecycle’: pay-as-you-go pensions. The transfers that pension contributors send (metaphorically) to fund their consumption as future pensioners are, technically, a legally underspecified claim on future consumption, to be financed later by younger others. In practice, pension contributions are used to finance pension benefits for today’s pensioners (older generations), with the expectation that these contributions establish some later claim on future consumption that will then have to be financed by contributions of future workers (younger generations). Pay-as-you-go schemes (states) and private saving or fully funded schemes (markets) are simply alternative mechanisms (‘promises’ versus ‘assets’) to organize claims on younger generations’ future output–to solve the consumption smoothing problem [18,26]. In both cases, lifecycle redistribution operates through cross-sectional reallocations between different age groups.

The particular solution offered by welfare *states* uses taxes and promises to exploit the sectional nature of the lifecycle. At any given point in time, people who have been born in different years (different cohorts) live together (as age groups) in the same society. Hence there are always net ‘resource productive’ people (typically the working-aged) who can be taxed to finance net transfers downward to children and upward to the elderly [27,28]. Welfare state entitlement claims are based not on classical legal contracts but rather on a ‘relational contract,’ a form of intertemporal trust that successive generations (or the future governments representing them) will ‘honor’ their ‘promise’ or ‘obligation’ [4,25,29]. The frequently observed changes over time in pension generosity or benefit formulas, for instance, are just a manifestation of a new political equilibrium between taxpayers and recipients due to changed economic-fiscal circumstances [30,31].

In other words, the welfare state solves the endemic problem of lifecycle consumption smoothing given inherently incomplete contracts about the future by arranging resource reallocations between age groups in cross-section. This article reconceptualizes the piggy bank function accordingly and then proceeds to empirically assess its importance relative to the Robin Hood function in a first-ever analysis of the *joint* distribution of socio-economic status, age and, respectively, (a) all cash and in-kind transfers (‘benefits’), (b) financing contributions (‘taxes’), and (c) resulting ‘net benefits.’ We investigate a sample of over 400,000 Europeans from 22 European Union member states: Belgium, Bulgaria, Cyprus, the Czech Republic, Germany, Denmark, Estonia, Finland, France, Greece, Hungary, Ireland, Latvia, Lithuania, Luxemburg, Poland, Portugal, Romania, Spain, Sweden, Slovakia, and the United Kingdom. These represent 82 percent of the European Union’s population and all major institutional welfare state types, in 2010. The article is organized as follows. We discuss our methodological approach in the next section. We then introduce our model and present the three-dimensional distribution surfaces of all cash and in-kind welfare benefits, the taxes that finance them, and the resulting net benefits for 22 European welfare states by SES and age. We then quantify the relative importance of the two explanatory variables in terms of their average marginal effects and the Shapley value of their contribution to the explained variance. The last section discusses wider implications for how to understand and evaluate social policy.

## Methodological approach: Separating Robin Hood and piggy bank in cross-section

Methodologically, social scientists have typically inferred the welfare state’s ‘lifecycle redistribution’ function from the difference between lifetime inequality and period inequality. If inequalities appear to be significantly smaller among lifetime incomes than they are in any cross-section, this is then inferred to be the result of a piggy bank in operation. Precisely such findings have been published for a handful of countries so far. The tax-transfer system has been found to entail significantly lower lifetime inequalities, as compared to period inequalities in the US [32], the UK [33–35], the Netherlands [36], Ireland [37], Germany [38], Australia [39] and Sweden [40,41]. Other studies discuss the difference between period and lifetime inequalities without relating it to tax-and-transfer systems [8,42].

This common methodological approach carries significant limitations, however. Direct cross-country comparison of results is difficult (rare examples are [43] and [44], both comparing only two countries). Measuring intertemporal effects requires a time-series of retrospective data, which is only available for a few countries. Even if such data are at hand, future lifecycle sections have to be simulated, implying numerous idiosyncratic assumptions and weakening comparability. More importantly, the design of separating the Robin Hood and piggy bank functions by comparing period and lifetime inequalities offers only indirect and incidental evidence. The observation that lifetime inequalities are smaller than period inequalities may not even be ascribable to the piggy bank role. In general, the measured level of inequality is sensitive to the length of the accounting period. As a general rule, the longer the period, the *lower* the inequalities. This is due, first, to the oscillation of individual incomes: the longer the accounting period (a year versus a week), the smaller the inequalities as there will be a reversal to one’s own mean [45]. It is due, second, to the well-established hump-shaped pattern of income over the lifecycle [46]. Consequently, lifetime inequalities can be assumed to be lower than cross-section inequalities, without any need to refer to how the piggy bank operates. In other words, one cannot adequately capture the piggy bank function based on a design that compares lifetime inequalities with cross-sectional inequalities.

Rather than engaging in single-country microsimulation over the lifecycle or in a meta-analysis of hard-to-compare country results, this article therefore approaches the two-function separation problem without recourse to intertemporal data. Estimating the relative role of inter-status and inter-age redistribution in determining welfare benefits, taxes, and net benefits requires the analysis of their respective *joint* distributions with both SES and age. Age and income (or wealth) have frequently been used simultaneously in the decomposition of *inequalities* since [46] first isolated the age component of inequality indexes [47]. The dependent variable of these decomposition models has typically been some measure of (income or status) inequality. But similar decomposition analyses using access to welfare benefits, taxes, and the resulting net welfare benefits as their dependent variables, as we do here, have to our best knowledge not been attempted. Many studies hold some measure of inequality or poverty as the dependent variable and separate the effects of various components of the welfare state (e.g., [48]), but here we are interested in keeping the incidence of taxes or transfers on the left-hand side of the equation. There are large bodies of empirical research on transfer incidence by income, age, household type, or other individual characteristics, but the overwhelming majority are *uni*-dimensional, measuring the effect of a single explanatory variable. Some studies analyze distributions in several dimensions, but they do it separately, one after the other (e.g., [49]), and only a few discuss the incidence of transfers and taxes using age and SES simultaneously, such as generational accounting by gender (e.g., [50]) or by education level (e.g., [51]).

We use Household Budget Surveys (HBS), the European Union Statistics on Income and Living Conditions (EU SILC), and the European Health Interview Survey (EHIS). S1 File describes our data, the assumptions made in the analysis, and definitions of cash and in-kind benefits, direct and indirect taxes, and our measure of socio-economic status. Our welfare ‘benefits’ cover the full range of what is commonly considered as the welfare state: all benefits included under the UN’s Classification of Functions of Government (COFOG) functions 7 (‘health’), 9 (‘education’), and 10 (‘social protection’) in the national accounting categories of ‘final consumption expenditures’ and ‘social benefits other than social transfers in-kind’ (all at the level of the general government). Effectively, the analysis covers the entire public spending category of ‘individual consumption expenditures,’ those for which specific beneficiaries can be assigned. The other COFOG categories (general public services, defense, public order and safety, economic affairs, environmental protection, housing and community amenities, and recreation, culture, and religion) finance ‘collective consumption expenditures’ or pure public goods that are age- and SES-neutral. Our measure of SES, based on the standard index used by the OECD [52], is a composite indicator of education, occupational status, and home possessions (material deprivation and housing) at the household level. The calculations were repeated using alternative SES measures (see S1A and S1B Table).

## Analysis: The relative importance of age and socio-economic status

We apply a multivariate regression framework to compare the relative importance of age and SES in explaining differences in the receipt of benefits, the taxes and social contributions financing them, and the resulting net benefits. The statistical literature differentiates between importance based on impact (change in the outcome variable in response to a unit change in the predictor variable) and dispersion (variance of the outcome variable explained by the regression equation that is attributable to a predictor variable). The former is measured using regression coefficients; the latter is based on changes in the *R*^*2*^ due to the stepwise inclusion or exclusion of predictor variables (e.g., [53]). In our basic specification, we apply ordinary least squares (OLS) regressions with age, status and their interaction on the right-hand side of the equation:

$$Y=\alpha +\sum_{i}\beta_{A_{i}}{Age}_{i}+\sum_{j}\beta_{S_{j}}{Status}_{j}+\sum_{i}\sum_{j}\beta_{I_{ij}}{Age}_{i}*{Status}_{j}+\varepsilon ,$$ (1)

where *Y* represents benefits, taxes, or net benefits, respectively, in the separate models; *Age*_*i*_ and *Status*_*j*_ are dummy variables representing categories of age and SES (*i*, *j* = 2,…, 10), and the *β*s are regression coefficients, so that *β*_*A*_s are the main effects of age *β*_*S*_s are the main effects for status and *β*_*I*_s are the interaction coefficients.

We use categorized versions of age and SES to allow for non-linearities in the effects of the variables. To avoid that the categorization of the two explanatory variables affects the between-group variation, we use age-deciles and status-deciles. This guarantees that the two marginal distributions are the same (see S2 Table). The interaction of age and SES is included in the model to allow for age and SES effects to differ by categories of the other variable. In the basic models presented here, we use only the two independent variables of interest. Control variables and country dummies are added in the robustness tests (see S2 File and S4 Table in S2 File). Our analysis does not aim for as complete an explanation as possible of the dependent variables. Rather, our purpose here is to compare the relative importance of age and SES by their regression coefficients and their contribution to the explained variance of the dependent variables. The correlation of age and SES is positive, but this affects only the standard errors of the estimates. Regression coefficient estimates remain unbiased in the presence of multicollinearity.

We pool the samples of 22 countries resulting in a European sample of over 400,000 individuals. As a first step, the national samples are reweighed so that each country is represented with the same number of people. Then, the national values for benefits and taxes are re-scaled in order to make them comparable. We follow the National Transfer Accounts method [54] and divide the national values of benefits and taxes by the national per capita labor income of the age-group 30–49. This re-scaling technique filters out the effect of differences in income level across countries, and it is less arbitrary than alternatives based on consumption baskets. The pooling procedure matches the corresponding national age-status groups. It preserves the properties of the national welfare systems with regard to access to benefits and contribution to their finances. However, it does not offer uniform age-brackets or status division lines. For easier interpretation, we refer to age-groups below by actual ages, but these are approximations based on simple country-averages (for details of sample pooling, see the S1 File). All results presented here are calculated from the pooled sample. We also repeated all calculations separately for each national sample and computed simple averages. This alternative method did not produce any substantive changes to our findings.

## Results

### Three-dimensional distribution surfaces

**Fig 1:**
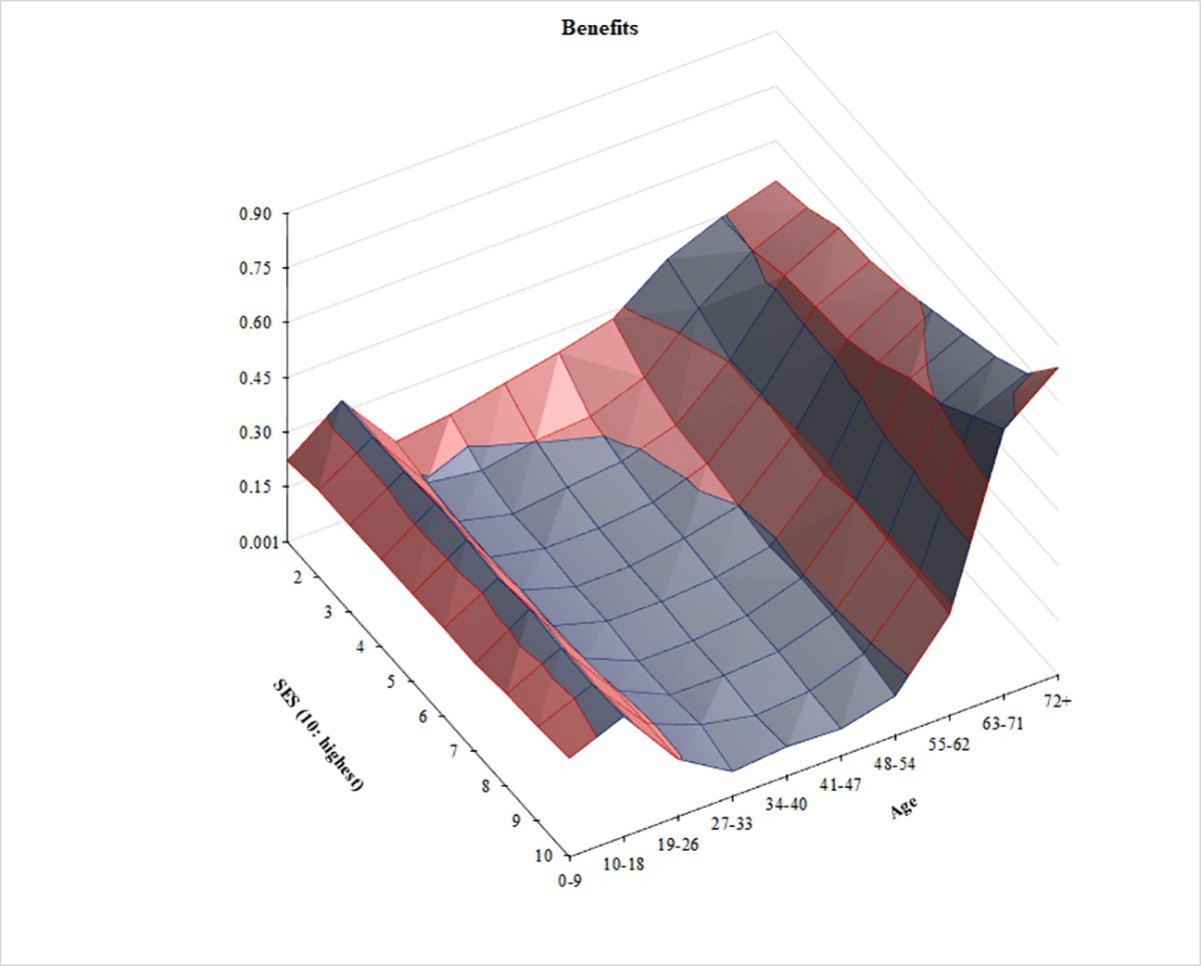
Per capita welfare benefits (cash and in-kind) by age and SES in the European Union. Source: Authors’ calculation. Notes: Calculated from a pooled sample of over 400,000 individuals from 22 EU countries. National values are re-scaled based on the average labor income of 30-49-year-olds. The image is rotated.

**Fig 2:**
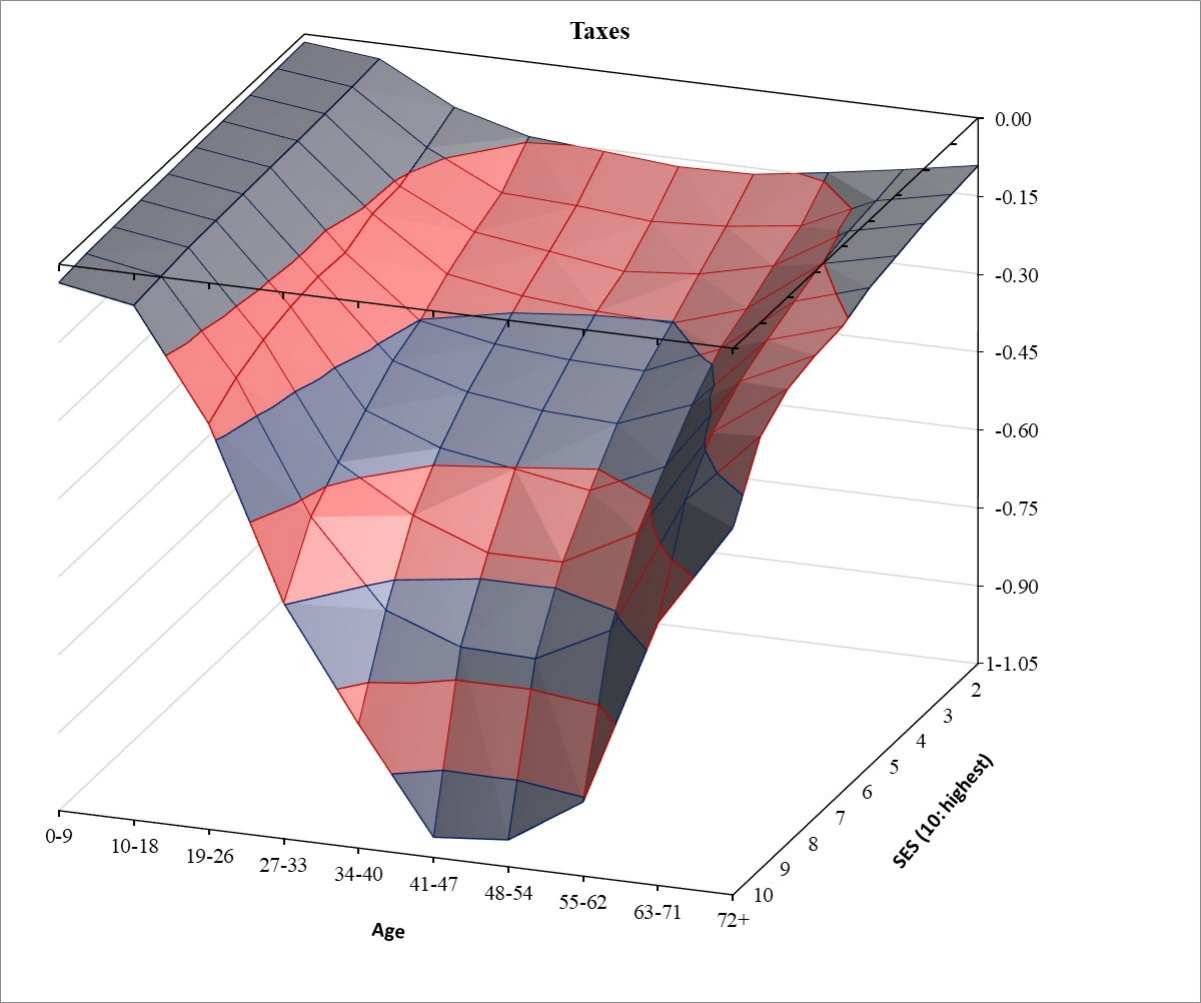
Per capita taxes and contributions financing welfare benefits by age and SES in the European Union. Sources and notes as in Fig 1.

**Fig 3:**
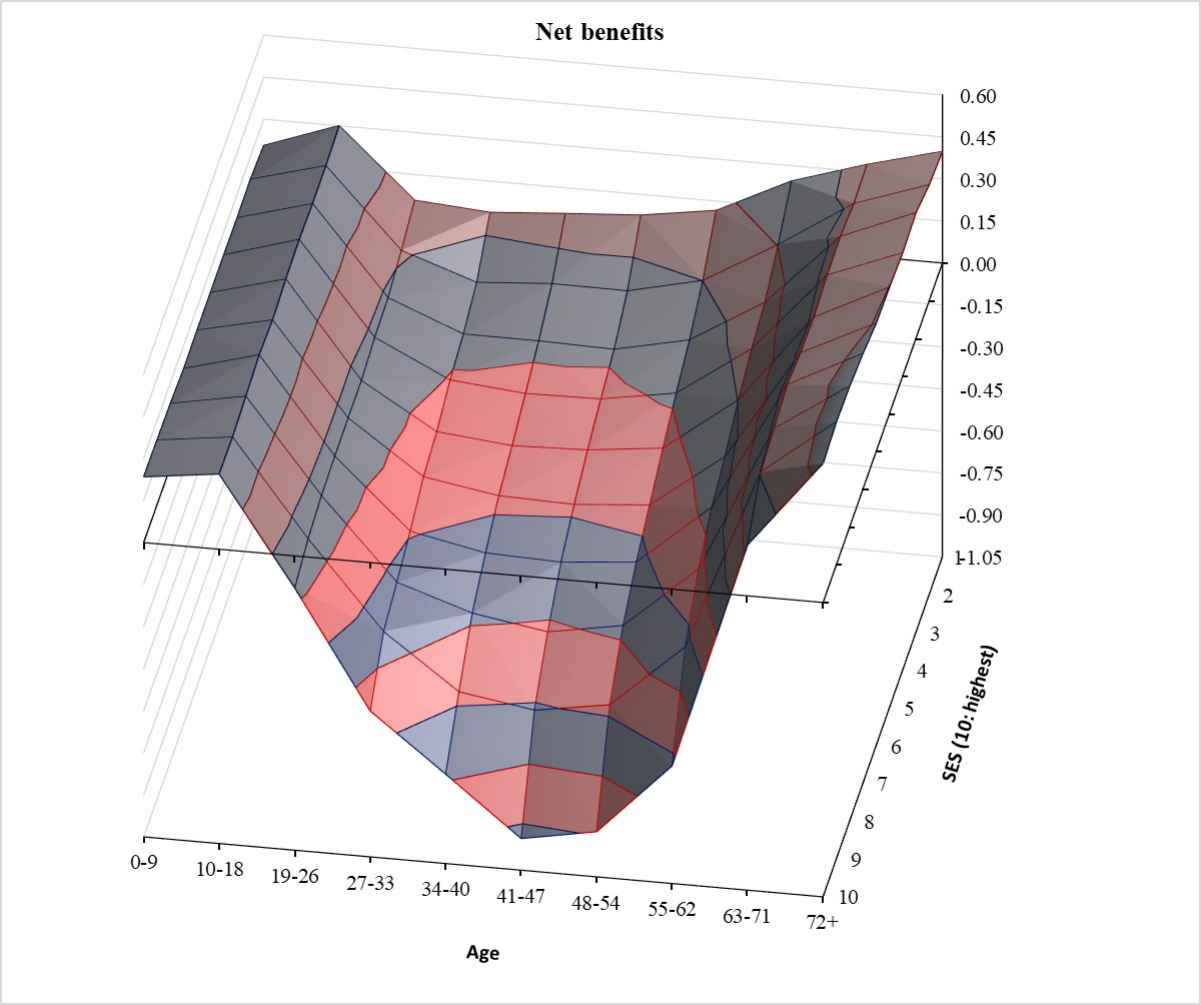
Per capita net welfare benefits (benefits minus taxes) by age and SES in the European Union. Sources and notes as in Fig 1.

Figs 1–3 show, respectively, the distributions of group averages for benefits, taxes, and net benefits in three-dimensional spaces. In each Fig, the two horizontal axes are age groups and SES categories. For better visibility, the charts are rotated. In addition, in S1 Fig we present these same findings in the form of two-dimensional line charts with separate lines for each of the ten SES groups. The full tables of the distribution surfaces for these group averages are presented in S3 Table. In addition, for completeness, tables of the regression surfaces drawn by the linear predictions are shown in S4A–S4C Table.

The relief map of welfare benefits (Fig 1) resembles a canyon with a river flowing downstream toward the reader: the right riverbank is on Fig 1‘s left, and vice versa. There is a slight upward right riverbank for the young, especially the second-youngest decile (roughly consisting of teenagers). Then there is a wide riverbed among the working-aged (deepest among the fifth to seventh age deciles), and a steep left riverbank among the elderly (three oldest deciles). The river flows entirely above-ground: all age groups and all status groups receive welfare benefits in Europe. Five notable things stand out visually. First, when it comes to welfare benefits receipt, age is more prominent than SES. Iso-age and iso-SES lines clearly illustrate this visually. With few exceptions, the iso-age lines run almost parallel with the SES axis. By contrast, the iso-status lines significantly deviate from running parallel to the age axis. In fact, second, the left riverbank rises both more steeply and higher: the oldest three age deciles receive significantly more welfare benefits than any of the younger deciles. In numerical terms, the average 10-to-18-year-old European receive more than three times as much as the average 27-to-33 and 34-to-40-year-old (the two age groups who receive the least). Meanwhile, the average person in the oldest age group gets almost six times as much as 27-to-40-year-olds. By contrast, among the SES groups, the largest difference is a mere 30% between the lowest and the highest values. In other words, when it comes to the transfer arm of the tax-and-transfer machinery, Europe consists of strongly elderly-oriented ‘benefit welfare states’ [55,56].

Third, there is only a minimal variance in the access to benefits by SES among children and the youth, especially among the 10-to-18-year-olds. All status groups receive similar welfare benefits; among the youngest children, there is even a slightly positive correlation between access and status. Fourth, the benefit side of European welfare states is, to a small extent, progressive among working-age people. The river flows downstream leisurely: after an initial cascade from the lowest to the second decile, its slope descends more gently from the second to the fifth decile among working-age people, and it becomes practically flat in the highest five SES deciles. However, fifth, the differences grow large and positively correlated with status in old age: we see a crease in the shape of the riverbank. Among the oldest-old, the highest-SES group receives 70 percent more benefits than the lowest-SES group.

Fig 2 reveals an altogether very different picture: not a canyon with a gently downstream flowing river, but a steep subterranean waterfall. European welfare states are distinctly progressive (redistributive across status) only through their *taxation* arm. This finding is a corollary to the result by Hills [34] for the UK, who showed that the lifecycle consumption financing aspect was considerably larger than the Robin Hood effect. The tax side in Fig 2 shows more substantial SES effects than the benefits side in Fig 1. Children only pay indirect taxes, which limits their contributions. Except for the two highest-status groups, more or less the same applies to the elderly. However, in contrast to the benefits side, here the iso-age lines also dip steeply in working age, especially in the highest SES decile. Yet even here, age matters crucially as well. As the iso-SES lines show, it is the working-aged who pay most taxes in every status group.

Lastly, Fig 3 visually represents the resulting *overall* picture of *net* welfare state benefits (benefits minus taxes) for our 22 European welfare states. We see a canyon, again with steep left and right riverbanks, but the river now goes underground and becomes yet steeper there. Five notable observations stand out. First, age dwarfs SES also in net terms. In each SES category, the oldest age group receives the most net welfare benefits (and the second oldest gets the second-largest sum except for the highest SES group). Second and third, once below ground, the river turns fast into a waterfall that cascades particularly steeply among the middle-aged higher SES groups. All age groups below 18 and all age groups above 63 are net welfare state recipients in every SES category. Fourth, in net terms, European welfare states are progressive, almost Rawlsian. In the lowest status category, all age groups are net beneficiaries. Fifth, the lowest status decile is the highest net beneficiary in all age groups between 10 and 62.

### Average marginal effects

**Fig 4:**
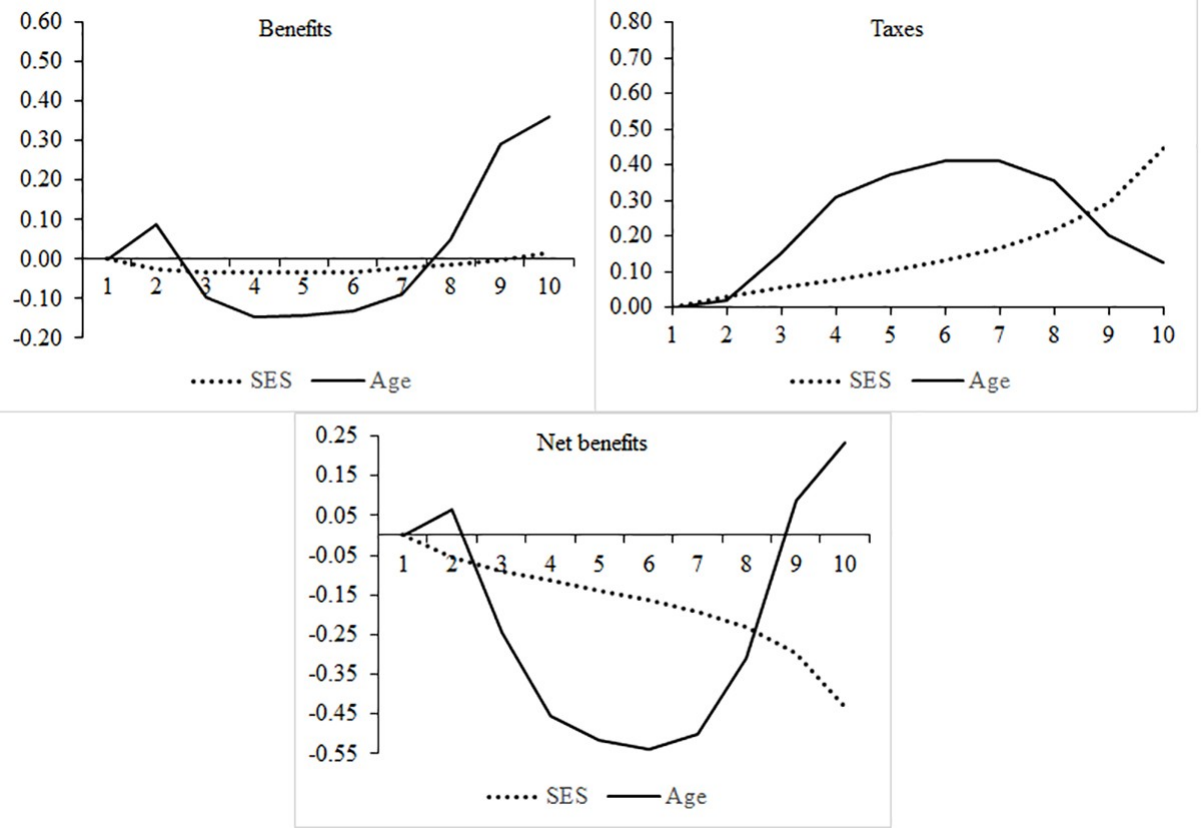
Average marginal effects of socio-economic status and age groups in the benefits, taxes, and net benefits models (reference category, age = 1 (youngest), SES = 1 (lowest)). Note: Based on regression models including only age and SES dummies and their interaction as explanatory variables.

The model in Eq (1) generates regression coefficients that draw regression surfaces similar to the distribution surfaces above. We present the series of age effects by status deciles and the series of status effects by age deciles in S4A–S4C Table. Here we focus only on the average marginal effects. In the three panels of Fig 4, we show the average marginal effects, respectively, of age across all status groups and of status across all age groups. The age coefficients, depicted by a solid line, go from the youngest age group (the reference category) to the oldest; the status coefficients (dotted line) go from the lowest status level (the reference category) to the highest. To help orientation, the scales of the vertical axes are the same, although the three panels depict different segments of the coordinate system. (For full regression tables for the three models, see S5 Table).

Fig 4 statistically confirms our previous visual results. European *benefits* states (upper left panel) are heavily elderly-oriented but almost neutral in terms of SES. *Tax* states (upper right panel) are progressive by SES, but, even more so, a burden on the middle-aged. Overall, in net terms (lower panel), European welfare states are status-redistributive still. But, more dominantly, they are a vehicle for taxing the middle-aged and financing the youngest (bottom two) age groups and, even more so, the elderly (oldest two) age groups.

Table 1 presents three indicators to summarize the differences in the coefficients of age and SES: the standard deviation, the sum of the absolute values, and the range of the coefficients. In the benefits model, the standard deviation of the SES coefficients is a mere 0.02; the sum of their absolute values is 0.21, and their range is 0.05 (see Table 1). The corresponding figures for age are 0.18, 1.38, and 0.50. In other words, on the benefit side of welfare states, age matters greatly, while SES does not.

**Table 1:** Sum of absolute values, range, and standard deviation of the average marginal effects of age and SES in models of benefits, taxes, and net benefits.

	Benefits		Taxes		Net benefits	
	SES	Age	SES	Age	SES	Age
Standard deviation	0.02	0.18	0.14	0.16	0.13	0.29
Sum of absolute values	0.21	1.38	1.53	2.36	1.71	2.95
Range	0.05	0.50	0.45	0.41	0.43	0.77

On the tax revenue side, both variables prove to be relevant, although age is clearly more important. On average, the age coefficients are further away from the reference category than are the SES coefficients, while their standard deviations and ranges are roughly similar. In the resulting net benefits model, lastly, age again is more important than SES. Net benefits differ more between age groups than between SES groups. All in all, European welfare states, as tax-and-transfer machines in net terms, redistribute more across age lines than across status lines.

### Explained variance

By the alternative interpretation of relative importance, a variable is more important than another if it explains more of the variance. Here, we use the Shapley method to study this version of variable importance [57]. In the Shapley-value decomposition, the contribution of an explanatory variable to the explained variance of the dependent variable is equal to this variable’s marginal effect on the goodness of fit of the model (*R*^2^). This marginal effect, in turn, is defined as the change in the *R*^2^ if the variable in question is eliminated from the regression. When there are several explanatory variables, the marginal effect of a variable depends on the order of elimination. The Shapley value of a regressor is the average of its marginal effects over all possible elimination orderings [53,58]. A particular advantage of the Shapley-method is that it decomposes the interaction term of two variables [58]. When the interaction of two variables is considered, the process of repeated eliminations includes the elimination of the interaction term, too. In this way, the contribution of the interaction term is split between the interacting variables, and the interaction term does not appear as a separate item in the regression table. The three-dimensional figures above suggest interactions between age and SES since the SES effect is visibly different in working age and old age.

We present the results of the decomposition exercise in Table 2. It contains percentages of the total variance of benefits, taxes, and net benefits, respectively, accounted for by age and SES, as well as the relative contributions of these variables to the variance explained just by the two of them. The analysis again confirms the conjectures of Section 2. Age is much more important in explaining access to welfare state benefits, and SES is in fact, irrelevant here. On the tax side, the two variables are both relatively important, but age still explains more of the variance. As for net welfare benefits, the analysis of the contributions to the explained variance gives a similar picture to the analysis of coefficients: age is more important, accounting for 24 percent of the total variance in net benefits explained (and 78 percent of the variance explained by both variables), compared to only 7 percent for socio-economic status (or 22 percent of joint variance).

**Table 2:** Contribution to the explained variance by age and SES for benefits, taxes, and net benefits (Shapley-value decomposition of the *R*^2^).

	Benefits		Taxes		Net benefits	
	Absolute contribution to *R*^*2*^	Relative contribution to *R*^*2*^ (%)	Absolute contribution to *R*^*2*^	Relative contribution to *R*^*2*^ (%)	Absolute contribution to *R*^*2*^	Relative contribution to *R*^*2*^ (%)
SES	1	5	10	43	7	22
Age	25	95	14	57	24	78
Total	26	100	25	100	31	100

Note: Absolute contributions sum to model R^2^. Relative contributions sum to 100%x.

These results are robust when the model is completed with additional controls for gender, household size, migration status, the degree of urbanization and country dummies. S6 Table presents similar summary statistics of the standardized regression coefficients as above (standard deviation and mean of the coefficients’ absolute values). The additional models show that age again dominates the benefits model, and SES again remains marginal. If any of our previous conclusions has to be modified, it is about the tax model. When gender and household size are included in the model, SES is even less on par with age. The latter is clearly more important in all three ways of measurement. It is also evident that none of the control variables is nearly as important as age.

In sum, the analysis based on explained variance confirms that European welfare states function as an intermediary between overlapping generations that seek to finance consumption over their lifecycle by exploiting the opportunities offered by that very overlap–contemporaries tend to be of different ages. More specifically still, welfare states serve as a channel through which working-age people of higher status support people of inactive age across all SES groups. Only the taxation arm of European welfare states, not their benefits arm, is distinctly progressive (redistributing strongly from high to low SES). Even here, redistribution across age groups is more important. But in terms of their *benefits* arm and, as a result, also in terms of the overall picture of *net benefits*, redistribution between age groups is clearly much more important than between socio-economic status groups.

## Conclusions and wider implications: How we evaluate social policies

European welfare states, first and foremost, are not status equalizers of sorts, but lifecycle redistribution machines in cross-section. This finding carries multiple wider implications for public policy. First, it affects the yardsticks we employ in targeting analysis. Far from limiting itself to socio-economic status, the analysis should be extended to targeting by age. How would this look? If the primary aim of social policy is to reallocate resources from working-age groups to children and the elderly, what does (mis-)targeting even mean? Let us again consider old-age pensions as an example. As a tool for mitigating poverty, public pension systems may (or may not) be effective, but they may also be, in a significant sense, inefficient, in that they overshoot. Pension benefits may raise the income of elderly people above the poverty line, but as an inter-age program, they may still be mistargeted if paid to people who are not yet old and/or whose productivity is still competitive. Effective retirement ages in our 22 EU countries have increased from 61.1 to 62.5 years for women and from 62.8 to 64.1 years for men between 2010 in 2018. This indicates a smaller degree of such mistargeting today, albeit with significant scope for improvement, especially in Eastern and Southern Europe.

Second, a perennially predominant question in contemporary social policy analysis–indeed, a key yardstick for judging ‘how successful are European welfare states’ [5]—is to what degree various welfare state types reduce poverty and inequality. Since Le Grand’s pioneering work [6] demonstrating the prevalence of mistargeting, it has become routine for national statistical services and international agencies such as the World Bank, the OECD or Eurostat to measure the distributional effects of welfare programs by income. In actual practice, higher-SES groups often receive as much or more in transfers and services than lower-SES groups–‘not-only-the-poor’ paradoxes or ‘Matthew effects’ [7,16,35], yet moving away from precise targeting by SES may paradoxically result in less, not more, poverty relief and inequality reduction ([59,60] but see [61,62]. Even social investment programs are not exempt from Matthew effects and may not much diminish inequality and poverty—a new ‘paradox of social investment’ [15,16]. However, our observation that welfare states are neither primarily nor solely responsible for poverty relief and income equalization should absolve or deflect some of the mistargeting and ineffectiveness blame leveled at them.

What may appear in univariate (‘SES’) models to be a dysfunctional Robin Hood state, badly targeted and riddled with Matthew effects, turns out in bivariate (‘SES-and-age’) models to be an inter-age-group resource reallocation state performing a *more* important second task rather well: lifecycle consumption smoothing. Well-meaning, even well-targeted, policies to reduce poverty or inequality in cross-section might lead to significant inter-cohort inequalities within societies, of the kind documented by [63] or [64]. Inequality measured in cross-section is always in part the result of age-specific, hump-shaped, productivity [20,32,65]. Hence, differences in the age composition of society, as captured in population pyramids, affect cross-sectional inequality irrespective of how welfare states operate.

Third, none of the above serves to question poverty alleviation and inequality reduction as worthy societal goals. Our finding that European welfare states are primarily inter-age reallocation machines does not imply, normatively, that public policies ought not to be used for poverty relief and inequality reduction. Rather, *other* forms of government activity—non-social policies—could also be drafted into that same societal effort and be judged accordingly. For example, road-construction projects are typically discussed exclusively in terms of technical efficiency. Yet infrastructure networks strongly impact equality, too: highways favor the residents of cities, where the rich live; but investing in lower-level road networks and public transport in the countryside brings greater benefits to the poor in relative terms. There is an abundance of similar examples: safety regulations, air pollution standards, public investment in air traffic or exchange rate policy, and carbon taxes, to name just a few [66]. The fuel tax increase announced by the Macron-Philippe government in France in 2018 led to massive and months-long *gilets jaunes* (yellow vests) protests for this reason. Though it was not interpreted as such by experts and politicians, this non-social policy was popularly perceived as having significant regressive effects–and resisted as such. These examples serve to illustrate the unspoken and unquestioned division of labor between various types of government intervention. Since they primarily operate as an inter-age reallocation system, social policies should not be singled out as the sole institution to shoulder the blame for imperfectly alleviating poverty and mitigating inequality. If these goals are deemed societally worthy, other non-social forms of government intervention could also be judged according to the same yardstick.

Fourth, our findings point to the need to reinterpret what welfare states mostly do. For many, welfare states are viewed as the primary remedy of poverty and inequalities. For others, they are a market-correcting institution, stepping in where markets fail, and to decommodify individuals. For yet others, they make individuals less resource-dependent on their families. This article does not take issue with these functions. Welfare states have evolved for multiple historical reasons to perform multiple functions. But our analysis does suggest, on empirical grounds, a shift in analytical focus. The underlying problem is not states *versus* markets and/or families. Esping-Andersen’s fundamental plea [11] for a new analytical focus on how states, markets and families *interact as triads* (regimes) to produce ‘welfare’ applies with equal force to lifecycle redistribution. Welfare states should primarily be viewed as an institutionalized way to solve a logically and historically prior problem confronting every member (irrespective of age, gender, or ideology) of every generation (irrespective of period) in every multi-generational society (irrespective of riches, welfare regime type, political economy model, or even age or degree of democracy): the fundamental lifecycle consumption financing problem [19,27].

All societies need to solve this problem because of the discrepancy between the bell curve of labor income and the flatter and more linear consumption curve over the lifecycle. *Welfare state* societies specifically solve it through the inter-age-group resource transfers of the piggy bank in cross-section: surplus resources are taxed away from the working-aged to finance childhood and old age. *European* welfare societies engage in a division of labor to solve the problem: they are elderly-oriented welfare states relying on strongly child-oriented families [67–69]. Our general result obtains everywhere, even though there are country-specific differences within Europe as can be expected. For instance, in the non-Anglo-Saxon part of our sample, taxes are concentrated in working age with a rather linear slope according to status that turns somewhat steeper in the highest status group. In the UK, this slope really becomes much steeper in the last status groups. And in Ireland, the highest status group is taxed markedly more than any other status groups. Nevertheless, the strong status effect is combined with a strong age effect in these Anglo-Saxon welfare states, too. Our results are empirical and pertain strictly speaking only to the 22 European societies studied here. Other societies solve the same lifecycle consumption smoothing problem otherwise. For instance, in the United States, markets dwarf government in financing old age [70]. While contemporary tax burdens on working-age people are unsurprisingly much higher in ‘statist’ Sweden compared to ‘familialist’ Taiwan, the combined weight of net public and net private transfers is nearly identical in both countries [28]. Swedish workers (and Europeans more generally) pay taxes to their government and trust it to provide for their parents; a heavily socialized solution. Taiwanese workers (and Confucian cultures more generally) provide for their own family members directly; a heavily familialized solution [71].

European welfare states solve the problem of lifecycle consumption smoothing given incomplete contracts about the future by, as it were, sequentially sidestepping the future. But of course, the shadow of the future looms large, in the form of ever-contingent power balances between successive generations over time. Younger generations must eternally follow older generations–and be willing, politically, to finance the latter’s consumption [29,72]. The key requirement for the continued functioning of any intertemporal redistribution vehicle is productivity-adjusted demographic continuity. As Samuelson put it [27: 482], pay-as-you-go schemes are a claim on ‘the community’s indestructible real tax base.’ At a fundamental level therefore, lifecycle consumption financing depends less on property rights or state versus market solutions than on how successive cohorts of voters use their relative political power. Future policy research should conceptualize intergenerational justice more consistently in terms of inter-cohort resource equality and policy sustainability. Theoretical research could fruitfully model political sustainability and the forward and backward linkages that bind overlapping cohorts [73–75]. For, in a very real sense, retirement reform must begin with babies [12] and social security is good for the environment [76]. In a final paradox, then, a clearer understanding of the cross-sectional operation of the piggy bank leads to a more urgent focus on time and the generations.

## Supporting information

S1 Fig Per capita welfare benefits (cash and in-kind), taxes and contributions, and net welfare benefits (benefits minus taxes) by age (horizontal axis) and SES (colored lines) in the European Union; two-dimensional figures. Notes: The three panels replicate Figs 1–3 of the main text in a two-dimensional line chart format. SES groups are numbered from 1 (lowest) to 10 (highest).
(TIF)

S1 Table. a. Standard deviation, sum of absolute values, and range of regression coefficients of age and SES for models of benefits, taxes and net benefits using alternative indicators of socio-economic status. Note: The table is based on regression models including age and SES dummies (but no interaction terms) as explanatory variables. b. Contribution to the explained variance by age and SES on benefits, taxes and net benefits (Shapley-value decomposition of the R2) using alternative indicators of socio-economic status. Note: Absolute contributions sum to model R2, while relative contributions sum to 100 (DOCX)

S2 Table Sample characteristics: Age deciles and SES deciles in the regression models: Group means and sample shares. Note: In order to keep the distribution of the sample among deciles as even as possible, we also took into account the quarters of the birth year.
(DOCX)

S3 Table Distribution surfaces: per capita welfare benefits, taxes and net benefits by age-group and SES decile in the pooled European sample. Note: Distribution surfaces presented as Figs 1–3 in the main text. National samples are reweighed so that each country is represented with the same number of people. The national values for benefits and taxes are then re-scaled following the National Transfer Accounts method (national values of benefits and taxes by the national per capita labor income of the age-group 30–49). The pooling procedure matches the corresponding national age-status groups: the oldest of one country are paired with the oldest of another and people of the lowest status in one country with people of the lowest status in another.
(DOCX)

S4 Table. a. Benefits model: age effects by status deciles and status effects by age deciles. b. Taxes model: age effects by status deciles and status effects by age deciles. c. Net benefits model: age effects by status deciles and status effects by age deciles (DOCX)

S5 Table Average marginal effects of socio-economic status and age groups in the benefits, taxes, and net benefits models: *β*s, *t*-statistics and *p*-values for models of benefits, taxes, and net benefits. Notes. Average marginal effects of socio-economic status and age groups in the benefits, taxes, and net benefits models as in Fig 4 in the main text.
(DOCX)

S6 Table Standard deviation and mean absolute value of regression coefficients and relative contribution to the explained variance for benefits, taxes, and net benefits by age, SES, country dummies and control variables. Notes: Absolute contributions sum to model *R*^*2*^, while relative contributions sum to 100%. Household size is added as a continuous variable, not as a group of dummies, so the standard deviation and the mean absolute values are not applicable. Migration status has three categories: non-migrant (the reference category), migrant from another EU-country, and migrant from beyond the EU. Urbanization also has three categories: densely populated (the reference category), intermediate, and thinly populated. We also added country dummies to the model, but no interaction terms are included. Note that instead of the sum, we use the mean of the absolute values because the variable-groups do not consist of the same number of variables. Besides, we present the results of the Shapley-value decomposition of the relative contributions to the explained variance.
(DOCX)

S7 Table. a. Standard deviation, sum of absolute values, and range of regression coefficients of age and SES for models of benefits, taxes and net benefits with alternative incidence assumptions. Note: Alternative incidence assumptions: parents, not the children, receive family benefits and pay taxes on children’s consumption. The table is based on regression models including age and SES dummies (but no interaction terms) as explanatory variables. b. Contribution to the explained variance by age and SES on benefits, taxes and net benefits (Shapley-value decomposition of the R2) with alternative incidence assumptions. Note: Alternative incidence assumptions: see above. The table is based on regression models including age and SES dummies (but no interaction terms) as explanatory variables. Absolute contributions sum to model R2, while relative contributions sum to 100 (DOCX)

S1 File Data, definitions, and assumptions. (DOCX)

S2 File Models with control variables and country dummies. (DOCX)
