# Supplementary figures and images for: Welfare states as lifecycle redistribution machines: Decomposing the roles of age and socio-economic status shows that European tax-and-benefit systems primarily redistribute across age groups
Source: PLoS One. 2021 Aug 25;16(8):e0255760. doi: 10.1371/journal.pone.0255760 (PMC8386825; doi:10.1371/journal.pone.0255760)

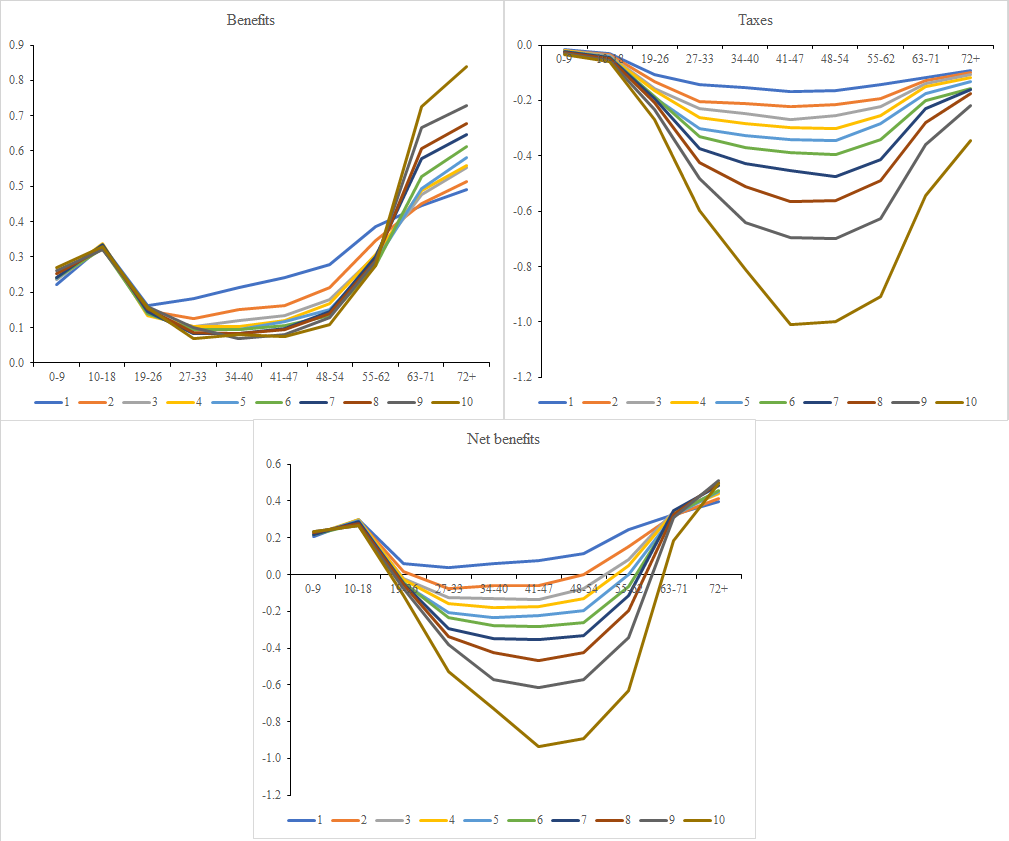

Supplement: S1 Fig — Notes: The three panels replicate Figs 1–3 of the main text in a two-dimensional line chart format. SES groups are numbered from 1 (lowest) to 10 (highest). (TIF) [file pone.0255760.s001.tif]
