# Supplementary material for: Welfare states as lifecycle redistribution machines: Decomposing the roles of age and socio-economic status shows that European tax-and-benefit systems primarily redistribute across age groups
Source: PLoS One. 2021 Aug 25;16(8):e0255760. doi: 10.1371/journal.pone.0255760 (PMC8386825; doi:10.1371/journal.pone.0255760)
Supplement: S1 Table. a. Standard deviation, sum of absolute values, and range of regression coefficients of age and SES for models of benefits, taxes and net benefits using alternative indicators of socio-economic status. Note: The table is based on regression models including age and SES dummies (but no inter — (DOCX) [file pone.0255760.s002.docx]

**S2 Table a. Standard deviation, sum of absolute values, and range of regression coefficients of age and SES for models of benefits, taxes and net benefits using alternative indicators of socio-economic status.**

|  | Benefits | | Taxes | | Net benefits | |
| --- | --- | --- | --- | --- | --- | --- |
|  | SES | Age | SES | Age | SES | Age |
| **SES indicator based on education, occupation, material living standards and housing** | | | | | | |
| Standard deviation | 0.01 | 0.16 | 0.09 | 0.11 | 0.06 | 0.14 |
| Sum of absolute values | 0.11 | 1.20 | 0.93 | 1.71 | 0.76 | 1.50 |
| Range | 0.03 | 0.41 | 0.29 | 0.30 | 0.21 | 0.38 |
| **SES indicator based on education, occupation and housing** | | | | | | |
| Standard deviation | 0.01 | 0.16 | 0.08 | 0.11 | 0.02 | 0.05 |
| Sum of absolute values | 0.10 | 1.21 | 0.82 | 1.73 | 0.25 | 0.56 |
| Range | 0.03 | 0.42 | 0.26 | 0.30 | 0.07 | 0.12 |
| **SES indicator based on education and occupation** | | | | | | |
| Standard deviation | 0.01 | 0.16 | 0.08 | 0.11 | 0.06 | 0.15 |
| Sum of absolute values | 0.17 | 1.21 | 0.82 | 1.74 | 0.53 | 1.51 |
| Range | 0.04 | 0.42 | 0.26 | 0.30 | 0.18 | 0.38 |

Note: The table is based on regression models including age and SES dummies (but no interaction terms) as explanatory variables.

**S2 Table b. Contribution to the explained variance by age and SES on benefits, taxes and net benefits (Shapley-value decomposition of the *R^2^*) using alternative indicators of socio-economic status.**

|  | Benefits | | Taxes | | Net benefits | |
| --- | --- | --- | --- | --- | --- | --- |
|  | Absolute | Relative | Absolute | Relative | Absolute | Relative |
|  | contribution to *R^2^* | | | | | |
| **SES indicator based on education, occupation, material living standards and housing** | | | | | | |
| SES | 0 | 1 | 8 | 41 | 5 | 18 |
| age | 24 | 99 | 12 | 59 | 22 | 82 |
| total | 24 | 100 | 20 | 100 | 27 | 100 |
| **SES indicator based on education, occupation and housing** | | | | | | |
| SES | 0 | 2 | 7 | 37 | 4 | 16 |
| age | 24 | 98 | 12 | 63 | 22 | 84 |
| total | 24 | 100 | 19 | 100 | 27 | 100 |
| **SES indicator based on education and occupation** | | | | | | |
| SES | 1 | 2 | 7 | 37 | 4 | 17 |
| age | 23 | 98 | 12 | 63 | 22 | 83 |
| total | 24 | 100 | 19 | 100 | 27 | 100 |

Note: Absolute contributions sum to model *R^2^*, while relative contributions sum to 100%.
