# Supplementary material for: Welfare states as lifecycle redistribution machines: Decomposing the roles of age and socio-economic status shows that European tax-and-benefit systems primarily redistribute across age groups
Source: PLoS One. 2021 Aug 25;16(8):e0255760. doi: 10.1371/journal.pone.0255760 (PMC8386825; doi:10.1371/journal.pone.0255760)
Supplement: S2 Table — Note: In order to keep the distribution of the sample among deciles as even as possible, we also took into account the quarters of the birth year. (DOCX) [file pone.0255760.s003.docx]

**S3 Table. Sample characteristics: Age deciles and SES deciles in the regression models: group means and sample shares.**

|  | Mean | Share of sample, % |
| --- | --- | --- |
|  | Age deciles (age in years) | |
| 1 | 5.2 | 10.3 |
| 2 | 14.7 | 10.0 |
| 3 | 22.8 | 10.0 |
| 4 | 30.3 | 10.0 |
| 5 | 37.4 | 10.0 |
| 6 | 44.3 | 10.1 |
| 7 | 51.4 | 9.9 |
| 8 | 58.5 | 9.9 |
| 9 | 67.0 | 10.0 |
| 10 | 77.8 | 9.8 |
|  | SES deciles (SES scores) | |
| 1 | -2.4 | 10.0 |
| 2 | -1.4 | 10.0 |
| 3 | -0.9 | 10.0 |
| 4 | -0.5 | 10.0 |
| 5 | -0.2 | 10.0 |
| 6 | 0.2 | 10.0 |
| 7 | 0.6 | 10.0 |
| 8 | 1.1 | 10.0 |
| 9 | 1.7 | 10.0 |
| 10 | 2.4 | 10.0 |

Note: In order to keep the distribution of the sample among deciles as even as possible, we also took into account the quarters of the birth year.
