# Supplementary material for: Welfare states as lifecycle redistribution machines: Decomposing the roles of age and socio-economic status shows that European tax-and-benefit systems primarily redistribute across age groups
Source: PLoS One. 2021 Aug 25;16(8):e0255760. doi: 10.1371/journal.pone.0255760 (PMC8386825; doi:10.1371/journal.pone.0255760)
Supplement: S3 Table — Note: Distribution surfaces presented as Figs 1–3 in the main text. National samples are reweighed so that each country is represented with the same number of people. The national values for benefits and taxes are then re-scaled following the National Transfer Accounts method (national values of benefits and taxes by the national per capita labor income of the age-group 30–49). The pooling procedure matches the corresponding national age-status groups: the oldest of one country are paired with the oldest of another and people of the lowest status in one country with people of the lowest status in another. (DOCX) [file pone.0255760.s004.docx]

**S6 Table. Distribution surfaces: per capita welfare benefits, taxes and net benefits by age-group and SES decile in the pooled European sample.**

|  | **Socio-economic status decile** | | | | | | | | | |
| --- | --- | --- | --- | --- | --- | --- | --- | --- | --- | --- |
|  | 1 | 2 | 3 | 4 | 5 | 6 | 7 | 8 | 9 | 10 |
| **Age decile** | **Benefits** | | | | | | | | | |
| 1 | 0.22 | 0.24 | 0.24 | 0.24 | 0.24 | 0.24 | 0.24 | 0.25 | 0.26 | 0.27 |
| 2 | 0.33 | 0.33 | 0.33 | 0.34 | 0.33 | 0.32 | 0.34 | 0.32 | 0.32 | 0.33 |
| 3 | 0.16 | 0.15 | 0.14 | 0.13 | 0.14 | 0.14 | 0.15 | 0.15 | 0.16 | 0.16 |
| 4 | 0.18 | 0.13 | 0.10 | 0.10 | 0.09 | 0.10 | 0.08 | 0.09 | 0.10 | 0.07 |
| 5 | 0.21 | 0.15 | 0.12 | 0.10 | 0.10 | 0.09 | 0.08 | 0.08 | 0.07 | 0.08 |
| 6 | 0.24 | 0.16 | 0.13 | 0.12 | 0.12 | 0.10 | 0.10 | 0.09 | 0.08 | 0.07 |
| 7 | 0.28 | 0.21 | 0.18 | 0.17 | 0.15 | 0.13 | 0.14 | 0.14 | 0.13 | 0.11 |
| 8 | 0.39 | 0.35 | 0.30 | 0.31 | 0.29 | 0.28 | 0.30 | 0.29 | 0.29 | 0.28 |
| 9 | 0.45 | 0.45 | 0.48 | 0.49 | 0.50 | 0.53 | 0.58 | 0.61 | 0.67 | 0.73 |
| 10 | 0.49 | 0.51 | 0.55 | 0.56 | 0.58 | 0.61 | 0.65 | 0.68 | 0.73 | 0.84 |
|  | **Taxes** | | | | | | | | | |
| 1 | -0.02 | -0.02 | -0.02 | -0.02 | -0.02 | -0.02 | -0.02 | -0.03 | -0.03 | -0.04 |
| 2 | -0.03 | -0.03 | -0.04 | -0.04 | -0.04 | -0.04 | -0.05 | -0.05 | -0.05 | -0.06 |
| 3 | -0.11 | -0.13 | -0.16 | -0.16 | -0.19 | -0.18 | -0.19 | -0.21 | -0.23 | -0.27 |
| 4 | -0.14 | -0.20 | -0.23 | -0.26 | -0.30 | -0.33 | -0.37 | -0.43 | -0.48 | -0.60 |
| 5 | -0.15 | -0.21 | -0.25 | -0.28 | -0.33 | -0.37 | -0.43 | -0.51 | -0.64 | -0.81 |
| 6 | -0.17 | -0.22 | -0.27 | -0.30 | -0.34 | -0.39 | -0.45 | -0.56 | -0.70 | -1.01 |
| 7 | -0.16 | -0.21 | -0.26 | -0.30 | -0.34 | -0.40 | -0.48 | -0.56 | -0.70 | -1.00 |
| 8 | -0.14 | -0.19 | -0.22 | -0.26 | -0.28 | -0.34 | -0.41 | -0.49 | -0.63 | -0.91 |
| 9 | -0.12 | -0.13 | -0.14 | -0.15 | -0.17 | -0.20 | -0.23 | -0.28 | -0.36 | -0.54 |
| 10 | -0.09 | -0.10 | -0.11 | -0.12 | -0.13 | -0.16 | -0.16 | -0.18 | -0.22 | -0.34 |
|  | **Net benefits** | | | | | | | | | |
| 1 | 0.21 | 0.22 | 0.22 | 0.22 | 0.22 | 0.22 | 0.22 | 0.23 | 0.23 | 0.23 |
| 2 | 0.30 | 0.29 | 0.29 | 0.30 | 0.29 | 0.28 | 0.29 | 0.28 | 0.27 | 0.27 |
| 3 | 0.06 | 0.01 | -0.02 | -0.03 | -0.05 | -0.05 | -0.05 | -0.05 | -0.07 | -0.11 |
| 4 | 0.04 | -0.08 | -0.13 | -0.16 | -0.21 | -0.23 | -0.29 | -0.34 | -0.38 | -0.53 |
| 5 | 0.06 | -0.06 | -0.13 | -0.18 | -0.23 | -0.28 | -0.35 | -0.43 | -0.57 | -0.73 |
| 6 | 0.08 | -0.06 | -0.13 | -0.18 | -0.22 | -0.28 | -0.36 | -0.47 | -0.61 | -0.94 |
| 7 | 0.12 | 0.00 | -0.08 | -0.13 | -0.19 | -0.26 | -0.33 | -0.42 | -0.57 | -0.89 |
| 8 | 0.25 | 0.15 | 0.08 | 0.05 | 0.00 | -0.07 | -0.11 | -0.20 | -0.34 | -0.63 |
| 9 | 0.33 | 0.32 | 0.34 | 0.34 | 0.32 | 0.33 | 0.35 | 0.33 | 0.31 | 0.18 |
| 10 | 0.40 | 0.41 | 0.45 | 0.44 | 0.45 | 0.46 | 0.48 | 0.50 | 0.51 | 0.50 |

Note: Distribution surfaces are presented as Figs 1,2 and 3 in the main text. National samples are reweighed so that each country is represented with the same number of people. The national values for benefits and taxes are then re-scaled following the National Transfer Accounts method (national values of benefits and taxes by the national per capita labor income of the age-group 30-49). The pooling procedure matches the corresponding national age-status groups: the oldest of one country are paired with the oldest of another and people of the lowest status in one country with people of the lowest status in another.
