# Supplementary material for: Welfare states as lifecycle redistribution machines: Decomposing the roles of age and socio-economic status shows that European tax-and-benefit systems primarily redistribute across age groups
Source: PLoS One. 2021 Aug 25;16(8):e0255760. doi: 10.1371/journal.pone.0255760 (PMC8386825; doi:10.1371/journal.pone.0255760)
Supplement: S4 Table. a. Benefits model: age effects by status deciles and status effects by age deciles. b. Taxes model: age effects by status deciles and status effects by age deciles. c. Net benefits model: age effects by status deciles and status effects by age deciles — (DOCX) [file pone.0255760.s005.docx]

**S7 Table a. Benefits model: age effects by status deciles and status effects by age deciles.**

| Age effects by status deciles | | | | | | | | | | | |
| --- | --- | --- | --- | --- | --- | --- | --- | --- | --- | --- | --- |
|  |  | status deciles | | | | | | | | | |
|  |  | 1 | 2 | 3 | 4 | 5 | 6 | 7 | 8 | 9 | 10 |
| Age | 1 | 0.00 | 0.00 | 0.00 | 0.00 | 0.00 | 0.00 | 0.00 | 0.00 | 0.00 | 0.00 |
|  | 2 | 0.11 | 0.09 | 0.09 | 0.09 | 0.09 | 0.09 | 0.09 | 0.08 | 0.07 | 0.06 |
|  | 3 | -0.07 | -0.10 | -0.11 | -0.11 | -0.10 | -0.10 | -0.09 | -0.10 | -0.09 | -0.10 |
|  | 4 | -0.05 | -0.11 | -0.14 | -0.14 | -0.15 | -0.15 | -0.16 | -0.17 | -0.17 | -0.20 |
|  | 5 | -0.03 | -0.10 | -0.13 | -0.14 | -0.15 | -0.15 | -0.16 | -0.17 | -0.19 | -0.19 |
|  | 6 | -0.01 | -0.08 | -0.12 | -0.12 | -0.13 | -0.14 | -0.15 | -0.17 | -0.18 | -0.19 |
|  | 7 | 0.03 | -0.04 | -0.07 | -0.08 | -0.10 | -0.11 | -0.11 | -0.12 | -0.13 | -0.16 |
|  | 8 | 0.13 | 0.08 | 0.06 | 0.05 | 0.03 | 0.04 | 0.05 | 0.03 | 0.01 | 0.00 |
|  | 9 | 0.20 | 0.20 | 0.22 | 0.24 | 0.25 | 0.28 | 0.32 | 0.35 | 0.40 | 0.46 |
|  | 10 | 0.24 | 0.25 | 0.29 | 0.30 | 0.33 | 0.35 | 0.38 | 0.40 | 0.47 | 0.58 |
| Status effects by age deciles | | | | | | | | | | | |
|  |  | age deciles | | | | | | | | | |
|  |  | 1 | 2 | 3 | 4 | 5 | 6 | 7 | 8 | 9 | 10 |
| Status | 1 | 0.00 | 0.00 | 0.00 | 0.00 | 0.00 | 0.00 | 0.00 | 0.00 | 0.00 | 0.00 |
|  | 2 | 0.01 | -0.01 | -0.02 | -0.05 | -0.05 | -0.06 | -0.06 | -0.04 | 0.01 | 0.02 |
|  | 3 | 0.01 | -0.01 | -0.02 | -0.08 | -0.09 | -0.09 | -0.09 | -0.06 | 0.03 | 0.06 |
|  | 4 | 0.01 | -0.01 | -0.02 | -0.08 | -0.10 | -0.10 | -0.10 | -0.07 | 0.04 | 0.07 |
|  | 5 | 0.01 | -0.01 | -0.02 | -0.09 | -0.10 | -0.11 | -0.12 | -0.09 | 0.06 | 0.09 |
|  | 6 | 0.01 | -0.01 | -0.02 | -0.09 | -0.11 | -0.12 | -0.13 | -0.08 | 0.09 | 0.12 |
|  | 7 | 0.01 | -0.01 | -0.01 | -0.10 | -0.12 | -0.13 | -0.13 | -0.07 | 0.13 | 0.15 |
|  | 8 | 0.02 | -0.01 | -0.01 | -0.10 | -0.12 | -0.13 | -0.13 | -0.08 | 0.17 | 0.18 |
|  | 9 | 0.03 | -0.01 | 0.01 | -0.09 | -0.13 | -0.14 | -0.14 | -0.09 | 0.23 | 0.26 |
|  | 10 | 0.04 | -0.01 | 0.01 | -0.11 | -0.13 | -0.14 | -0.16 | -0.09 | 0.29 | 0.37 |

**S7 Table b. Taxes model: age effects by status deciles and status effects by age deciles.**

| Age effects by status deciles | | | | | | | | | | | |
| --- | --- | --- | --- | --- | --- | --- | --- | --- | --- | --- | --- |
|  |  | status deciles | | | | | | | | | |
|  |  | 1 | 2 | 3 | 4 | 5 | 6 | 7 | 8 | 9 | 10 |
| Age | 1 | 0.00 | 0.00 | 0.00 | 0.00 | 0.00 | 0.00 | 0.00 | 0.00 | 0.00 | 0.00 |
|  | 2 | -0.01 | -0.02 | -0.02 | -0.02 | -0.02 | -0.02 | -0.02 | -0.02 | -0.02 | -0.03 |
|  | 3 | -0.09 | -0.11 | -0.13 | -0.14 | -0.16 | -0.15 | -0.16 | -0.17 | -0.18 | -0.21 |
|  | 4 | -0.13 | -0.18 | -0.21 | -0.24 | -0.28 | -0.30 | -0.35 | -0.40 | -0.46 | -0.57 |
|  | 5 | -0.13 | -0.19 | -0.23 | -0.27 | -0.31 | -0.34 | -0.40 | -0.49 | -0.60 | -0.79 |
|  | 6 | -0.15 | -0.20 | -0.25 | -0.28 | -0.32 | -0.37 | -0.43 | -0.53 | -0.65 | -0.97 |
|  | 7 | -0.14 | -0.19 | -0.23 | -0.28 | -0.32 | -0.37 | -0.44 | -0.53 | -0.68 | -0.97 |
|  | 8 | -0.12 | -0.17 | -0.20 | -0.23 | -0.26 | -0.32 | -0.39 | -0.46 | -0.60 | -0.86 |
|  | 9 | -0.09 | -0.10 | -0.11 | -0.13 | -0.15 | -0.17 | -0.20 | -0.26 | -0.34 | -0.49 |
|  | 10 | -0.07 | -0.07 | -0.08 | -0.09 | -0.10 | -0.12 | -0.13 | -0.14 | -0.19 | -0.29 |
| Status effects by age deciles | | | | | | | | | | | |
|  |  | age deciles | | | | | | | | | |
|  |  | 1 | 2 | 3 | 4 | 5 | 6 | 7 | 8 | 9 | 10 |
| status | 1 | 0.00 | 0.00 | 0.00 | 0.00 | 0.00 | 0.00 | 0.00 | 0.00 | 0.00 | 0.00 |
|  | 2 | 0.00 | 0.00 | -0.03 | -0.06 | -0.06 | -0.05 | -0.06 | -0.05 | -0.01 | -0.01 |
|  | 3 | 0.00 | -0.01 | -0.05 | -0.09 | -0.10 | -0.10 | -0.10 | -0.08 | -0.03 | -0.01 |
|  | 4 | 0.00 | -0.01 | -0.06 | -0.12 | -0.14 | -0.13 | -0.15 | -0.12 | -0.04 | -0.03 |
|  | 5 | -0.01 | -0.01 | -0.08 | -0.16 | -0.18 | -0.17 | -0.19 | -0.15 | -0.07 | -0.04 |
|  | 6 | -0.01 | -0.01 | -0.07 | -0.18 | -0.22 | -0.23 | -0.24 | -0.21 | -0.09 | -0.06 |
|  | 7 | -0.01 | -0.02 | -0.08 | -0.23 | -0.28 | -0.29 | -0.31 | -0.28 | -0.12 | -0.07 |
|  | 8 | -0.01 | -0.02 | -0.09 | -0.29 | -0.36 | -0.39 | -0.40 | -0.35 | -0.18 | -0.09 |
|  | 9 | -0.01 | -0.02 | -0.11 | -0.34 | -0.48 | -0.52 | -0.56 | -0.49 | -0.26 | -0.13 |
|  | 10 | -0.02 | -0.03 | -0.14 | -0.47 | -0.67 | -0.84 | -0.85 | -0.76 | -0.42 | -0.24 |

**S7 Table c. Net benefits model: age effects by status deciles and status effects by age deciles.**

| Age effects by status deciles | | | | | | | | | | | |
| --- | --- | --- | --- | --- | --- | --- | --- | --- | --- | --- | --- |
|  |  | status deciles | | | | | | | | | |
|  |  | 1 | 2 | 3 | 4 | 5 | 6 | 7 | 8 | 9 | 10 |
| Age | 1 | 0.00 | 0.00 | 0.00 | 0.00 | 0.00 | 0.00 | 0.00 | 0.00 | 0.00 | 0.00 |
|  | 2 | 0.09 | 0.08 | 0.07 | 0.07 | 0.07 | 0.07 | 0.07 | 0.05 | 0.04 | 0.04 |
|  | 3 | -0.16 | -0.21 | -0.24 | -0.25 | -0.26 | -0.25 | -0.25 | -0.27 | -0.28 | -0.31 |
|  | 4 | -0.18 | -0.29 | -0.35 | -0.39 | -0.43 | -0.45 | -0.51 | -0.57 | -0.63 | -0.77 |
|  | 5 | -0.17 | -0.29 | -0.36 | -0.41 | -0.46 | -0.50 | -0.56 | -0.66 | -0.79 | -0.98 |
|  | 6 | -0.16 | -0.28 | -0.36 | -0.40 | -0.45 | -0.51 | -0.58 | -0.69 | -0.84 | -1.16 |
|  | 7 | -0.10 | -0.23 | -0.30 | -0.36 | -0.42 | -0.47 | -0.54 | -0.65 | -0.82 | -1.13 |
|  | 8 | 0.01 | -0.08 | -0.14 | -0.18 | -0.23 | -0.28 | -0.34 | -0.43 | -0.59 | -0.86 |
|  | 9 | 0.11 | 0.10 | 0.11 | 0.11 | 0.10 | 0.11 | 0.12 | 0.10 | 0.06 | -0.03 |
|  | 10 | 0.18 | 0.18 | 0.22 | 0.21 | 0.23 | 0.23 | 0.25 | 0.26 | 0.28 | 0.29 |
| Status effects by age deciles | | | | | | | | | | | |
|  |  | age deciles | | | | | | | | | |
|  |  | 1 | 2 | 3 | 4 | 5 | 6 | 7 | 8 | 9 | 10 |
| status | 1 | 0.00 | 0.00 | 0.00 | 0.00 | 0.00 | 0.00 | 0.00 | 0.00 | 0.00 | 0.00 |
|  | 2 | 0.01 | -0.01 | -0.04 | -0.11 | -0.12 | -0.11 | -0.12 | -0.09 | -0.01 | 0.01 |
|  | 3 | 0.01 | -0.02 | -0.07 | -0.17 | -0.19 | -0.19 | -0.19 | -0.15 | 0.00 | 0.05 |
|  | 4 | 0.01 | -0.01 | -0.08 | -0.21 | -0.24 | -0.23 | -0.25 | -0.19 | 0.00 | 0.04 |
|  | 5 | 0.00 | -0.02 | -0.10 | -0.25 | -0.29 | -0.28 | -0.31 | -0.23 | -0.01 | 0.05 |
|  | 6 | 0.00 | -0.02 | -0.09 | -0.27 | -0.33 | -0.35 | -0.37 | -0.29 | 0.00 | 0.05 |
|  | 7 | 0.00 | -0.03 | -0.09 | -0.33 | -0.40 | -0.42 | -0.44 | -0.35 | 0.01 | 0.08 |
|  | 8 | 0.01 | -0.03 | -0.10 | -0.39 | -0.48 | -0.52 | -0.54 | -0.43 | 0.00 | 0.10 |
|  | 9 | 0.02 | -0.03 | -0.10 | -0.43 | -0.61 | -0.66 | -0.70 | -0.58 | -0.03 | 0.12 |
|  | 10 | 0.02 | -0.04 | -0.13 | -0.58 | -0.80 | -0.98 | -1.01 | -0.85 | -0.12 | 0.13 |
