# Supplementary material for: Welfare states as lifecycle redistribution machines: Decomposing the roles of age and socio-economic status shows that European tax-and-benefit systems primarily redistribute across age groups
Source: PLoS One. 2021 Aug 25;16(8):e0255760. doi: 10.1371/journal.pone.0255760 (PMC8386825; doi:10.1371/journal.pone.0255760)
Supplement: S5 Table — Notes. Average marginal effects of socio-economic status and age groups in the benefits, taxes, and net benefits models as in Fig 4 in the main text. (DOCX) [file pone.0255760.s006.docx]

**S8 Table. Average marginal effects of socio-economic status and age groups in the benefits, taxes, and net benefits models: *β*s, *t*-statistics and *p*-values for models of benefits, taxes, and net benefits.**

|  | | Dependent variable: | | | Dependent variable: | | | Dependent variable: | | |
| --- | --- | --- | --- | --- | --- | --- | --- | --- | --- | --- |
|  |  | Benefits | | | Taxes | | | Net benefits | | |
|  |  | *β* | *t* | *p>t* | *β* | *t* | *p>t* | *β* | *t* | *p>t* |
| Age groups | 1 | 0.000 |  |  | 0.000 |  |  | 0.000 |  |  |
|  | 2 | 0.086 | 41.58 | 0.000 | 0.020 | 7.36 | 0.000 | 0.066 | 19.08 | 0.000 |
|  | 3 | -0.096 | -44.78 | 0.000 | 0.149 | 52.39 | 0.000 | -0.246 | -68.57 | 0.000 |
|  | 4 | -0.145 | -64.70 | 0.000 | 0.311 | 104.49 | 0.000 | -0.456 | -121.97 | 0.000 |
|  | 5 | -0.141 | -64.77 | 0.000 | 0.374 | 129.12 | 0.000 | -0.515 | -141.61 | 0.000 |
|  | 6 | -0.129 | -61.97 | 0.000 | 0.410 | 147.81 | 0.000 | -0.539 | -154.80 | 0.000 |
|  | 7 | -0.088 | -42.49 | 0.000 | 0.411 | 149.25 | 0.000 | -0.499 | -144.21 | 0.000 |
|  | 8 | 0.048 | 23.58 | 0.000 | 0.357 | 131.17 | 0.000 | -0.308 | -90.10 | 0.000 |
|  | 9 | 0.291 | 143.31 | 0.000 | 0.203 | 75.38 | 0.000 | 0.088 | 26.13 | 0.000 |
|  | 10 | 0.359 | 167.20 | 0.000 | 0.127 | 44.44 | 0.000 | 0.233 | 65.02 | 0.000 |
| SES groups | 1 | 0.000 |  |  | 0.000 |  |  | 0.000 |  |  |
|  | 2 | -0.024 | -11.30 | 0.000 | 0.032 | 11.52 | 0.000 | -0.056 | -16.06 | 0.000 |
|  | 3 | -0.032 | -15.43 | 0.000 | 0.057 | 20.45 | 0.000 | -0.089 | -25.67 | 0.000 |
|  | 4 | -0.034 | -16.32 | 0.000 | 0.078 | 28.31 | 0.000 | -0.113 | -32.47 | 0.000 |
|  | 5 | -0.035 | -16.79 | 0.000 | 0.103 | 37.29 | 0.000 | -0.139 | -39.92 | 0.000 |
|  | 6 | -0.032 | -15.38 | 0.000 | 0.131 | 47.29 | 0.000 | -0.164 | -47.01 | 0.000 |
|  | 7 | -0.023 | -11.00 | 0.000 | 0.168 | 60.30 | 0.000 | -0.191 | -54.73 | 0.000 |
|  | 8 | -0.015 | -7.37 | 0.000 | 0.217 | 77.71 | 0.000 | -0.232 | -66.40 | 0.000 |
|  | 9 | -0.002 | -1.02 | 0.308 | 0.293 | 104.54 | 0.000 | -0.296 | -83.95 | 0.000 |
|  | 10 | 0.015 | 7.04 | 0.000 | 0.447 | 157.33 | 0.000 | -0.433 | -121.20 | 0.000 |

Notes. Average marginal effects of socio-economic status and age groups in the benefits, taxes, and net benefits models as in Fig 4 in the main text.
