# Supplementary material for: Welfare states as lifecycle redistribution machines: Decomposing the roles of age and socio-economic status shows that European tax-and-benefit systems primarily redistribute across age groups
Source: PLoS One. 2021 Aug 25;16(8):e0255760. doi: 10.1371/journal.pone.0255760 (PMC8386825; doi:10.1371/journal.pone.0255760)
Supplement: S6 Table — Notes: Absolute contributions sum to model R2, while relative contributions sum to 100%. Household size is added as a continuous variable, not as a group of dummies, so the standard deviation and the mean absolute values are not applicable. Migration status has three categories: non-migrant (the reference category), migrant from another EU-country, and migrant from beyond the EU. Urbanization also has three categories: densely populated (the reference category), intermediate, and thinly populated. We also added country dummies to the model, but no interaction terms are included. Note that instead of the sum, we use the mean of the absolute values because the variable-groups do not consist of the same number of variables. Besides, we present the results of the Shapley-value decomposition of the relative contributions to the explained variance. (DOCX) [file pone.0255760.s007.docx]

**S9 Table. Standard deviation and mean absolute value of regression coefficients and relative contribution to the explained variance for benefits, taxes, and net benefits by age, SES, country dummies and control variables.**

|  | Standard deviation | | | Mean absolute values | | | Relative contribution to *R^2^* (%) | | |
| --- | --- | --- | --- | --- | --- | --- | --- | --- | --- |
|  | Benefits | Taxes | Net benefits | Benefits | Taxes | Net benefits | Benefits | Taxes | Net benefits |
| Age | 0.15 | 0.11 | 0.14 | 0.12 | 0.17 | 0.15 | 83 | 51 | 80 |
| SES | 0.01 | 0.08 | 0.06 | 0.02 | 0.09 | 0.07 | 1 | 33 | 17 |
| Gender | 0.04 | 0.07 | 0.03 | 0.03 | 0.05 | 0.02 | 1 | 4 | 1 |
| Household size | - | - | - | - | - | - | 7 | 2 | 0 |
| Migration status | 0.01 | 0.01 | 0.01 | 0.01 | 0.01 | 0.00 | 0 | 1 | 0 |
| Urbanization | 0.01 | 0.02 | 0.00 | 0.01 | 0.02 | 0.01 | 0 | 2 | 1 |
| Country | 0.03 | 0.03 | 0.01 | 0.02 | 0.02 | 0.01 | 8 | 6 | 1 |

Notes: Absolute contributions sum to model R^2^, while relative contributions sum to 100%. Household size is added as a continuous variable, not as a group of dummies, so the standard deviation and the mean absolute values are not applicable. Migration status has three categories: non-migrant (the reference category), migrant from another EU-country, and migrant from beyond the EU. Urbanization also has three categories: densely populated (the reference category), intermediate, and thinly populated. We also added country dummies to the model, but no interaction terms are included. Note that instead of the sum, we use the mean of the absolute values because the variable-groups do not consist of the same number of variables. Besides, we present the results of the Shapley-value decomposition of the relative contributions to the explained variance.
