# Supplementary material for: Welfare states as lifecycle redistribution machines: Decomposing the roles of age and socio-economic status shows that European tax-and-benefit systems primarily redistribute across age groups
Source: PLoS One. 2021 Aug 25;16(8):e0255760. doi: 10.1371/journal.pone.0255760 (PMC8386825; doi:10.1371/journal.pone.0255760)
Supplement: S7 Table. a. Standard deviation, sum of absolute values, and range of regression coefficients of age and SES for models of benefits, taxes and net benefits with alternative incidence assumptions. Note: Alternative incidence assumptions: parents, not the children, receive family benefits and pay taxe — (DOCX) [file pone.0255760.s008.docx]

**S10 Table a. Standard deviation, sum of absolute values, and range of regression coefficients of age and SES for models of benefits, taxes and net benefits with alternative incidence assumptions.**

|  | Benefits | | Taxes | | Net benefits | |
| --- | --- | --- | --- | --- | --- | --- |
|  | SES | Age | SES | Age | SES | Age |
| Standard deviation | 0.01 | 0.15 | 0.09 | 0.12 | 0.06 | 0.14 |
| Sum of absolute values | 0.15 | 1.17 | 0.91 | 1.84 | 0.78 | 1.30 |
| Range | 0.04 | 0.40 | 0.28 | 0.32 | 0.21 | 0.37 |

*Note: Alternative incidence assumptions: parents, not the children, receive family benefits and pay taxes on children’s consumption. The table is based on regression models including age and SES dummies (but no interaction terms) as explanatory variables.*

**S10 Table b. Contribution to the explained variance by age and SES on benefits, taxes and net benefits (Shapley-value decomposition of the *R^2^*) with alternative incidence assumptions.**

|  | Benefits | | Taxes | | Net benefits | |
| --- | --- | --- | --- | --- | --- | --- |
|  | Absolute | Relative | Absolute | Relative | Absolute | Relative |
|  | contribution to *R^2^* | | | | | |
| SES | 0 | 1 | 8 | 37 | 5 | 21 |
| Age | 22 | 99 | 14 | 63 | 20 | 79 |
| Total | 22 | 100 | 22 | 100 | 25 | 100 |

*Note: Alternative incidence assumptions: see above. The table is based on regression models including age and SES dummies (but no interaction terms) as explanatory variables. Absolute contributions sum to model R^2^, while relative contributions sum to 100%.*
