# Supplementary material for: Welfare states as lifecycle redistribution machines: Decomposing the roles of age and socio-economic status shows that European tax-and-benefit systems primarily redistribute across age groups
Source: PLoS One. 2021 Aug 25;16(8):e0255760. doi: 10.1371/journal.pone.0255760 (PMC8386825; doi:10.1371/journal.pone.0255760)
Supplement: S1 File — (DOCX) [file pone.0255760.s009.docx]

# S1 Data, definitions, and assumptions

## Data

The primary data sources are Household Budget Surveys (HBS) and the European Union Statistics on Income and Living Conditions (EU SILC). As a complement, the European Health Interview Survey is used to provide data on the consumption of healthcare services. We use the international version of the datasets, which usually offer less information than the original national surveys, to facilitate comparative analysis. The reference year is 2010, the last year for which comparable HBS data were available when writing the paper.

## Pooling the national samples

We pool the samples of 22 countries, Belgium, Bulgaria, Cyprus, the Czech Republic, Germany, Denmark, Estonia, Finland, France, Greece, Hungary, Ireland, Latvia, Lithuania, Luxemburg, Poland, Portugal, Romania, Spain, Sweden, Slovakia, and the United Kingdom, resulting in a European sample of over 400,000 individuals. The national samples are reweighed so that each country is represented with the same number of people. The national values for benefits and taxes are re-scaled based on the National Transfer Accounts method (1): the national values of benefits and taxes are divided by the national per capita labor income of the age group 30-49. This re-scaling technique filters out the effect of differences in income level across countries, and it is less arbitrary than alternatives based on consumption baskets. The pooling procedure matches the corresponding national age-status groups. This method pairs the oldest of one country with the oldest of another and people of the lowest status in one country with people of the lowest status in another one. It combines the national experiences of access to welfare benefits and contribution to their finances without being exposed to cross-country differences of age composition and status distribution. Besides, it preserves the desirable property of equal marginal group sizes. However, it does not offer uniform age brackets or status division lines. So, while it is certain that the oldest ten percent of each country belongs to the oldest age group of the matched sample, it is not guaranteed that people of the same age but different countries are in the same age bracket.

## Cash benefits

Cash benefits are recorded in several aggregate variables in EU-SILC, such as old-age pensions, survivor benefits, sickness benefits, disability benefits, unemployment benefits, education-related benefits, family/children related allowances, social exclusion benefits, and housing allowances. Most of these categories comprise several allowances.

Allocating cash benefits among household members can sometimes be less than straightforward. In some cases, such as family benefits, social exclusion benefits, and housing allowances, EU-SILC only includes household-level data, so assumptions need to be made when calculating individual-level benefits. In other cases, conceptual problems of incidence require intervention by the researcher. Particularly in countries with extended maternity and family benefits programs, the outcome of the analysis is affected by the choice of whether such benefits are assigned to the child or the parent. We made the analysis in both ways and allocated the benefits in question first to children (as our basic specification) then to their parents to check the robustness of the results (see S10 Table a-b below). The other household-level benefits (social exclusion, housing) are distributed uniformly among the household’s working-age adults.

Access to various social assistance-type benefits is frequently easier if children are present in the applicant’s household. We could not go into such detail and therefore assigned all such benefits to the applicant; this affects our conclusions in a conservative way by diminishing the effect of age on social policy.

## In-kind benefits

In contrast to cash benefits, the consumption of welfare-related public services in kind is not recorded in these surveys. We have to estimate the value – and sometimes the very use – of such services from external information sources. We used the assumption most frequently applied in the literature that a service’s value equals the average cost of its provision (2). Admittedly, this assumption ignores variations in the quality of public education and health care, which tend to be skewed in favor of the upper-middle class in many countries. This omission shows the status-related redistribution smaller than it is in reality if the overall direction of inter-status redistribution is from the poor to the rich; but it shows it larger if the overall inter-status redistribution is from the rich to the poor, which is the case in this paper. So, the omission of the quality component affects the results in a conservative way. Also, due to the limitations of the survey information, we cannot separate consumers of publicly and privately financed services.

As for education – both early childhood care and schooling – users can be identified from EU-SILC. The survey explicitly asks about attendance and the level of attendance for each household member. We imputed public spending by the user of the corresponding education and care levels. For health care, we applied the ‘insurance value approach’ (2), which assumes that every individual receives a benefit determined by the average healthcare spending on their group, irrespective of the services’ actual use. We employed data from the first wave of the European Health Interview Survey (EHIS), which records the number of days spent in hospital and the number of visits to a general practitioner or specialist. We calculated the average use of primary and outpatient services by gender, age group, and educational category, imputed these averages into the EU-SILC dataset, and weighted them by public spending per patient in the relevant service categories. Accordingly, our health profiles are based on frequency and length of medical care but not on actual spending. This simplification hides part of the cost variation since the worse a patient’s health is, the more expensive the intervention is. Once again, our results are distorted in a conservative way due to the generally poorer health of older people. Actual spending figures would be even more likely to be tilted towards the elderly.

## Macro adjustment of the benefit profile

The age-SES profiles of the cash and in-kind benefits received by individuals are adjusted to macro-aggregates of the European System of Accounts (Eurostat *gov_10a_exp*).

Instead of total expenditures, which include age- and SES-neutral public good type of items, such as capital transfers and gross capital formation in public health care, education, and social protection, we apply the sum of social benefits other than social transfers in kind (for cash transfers) and final consumption expenditures (for benefits in kind) excluding benefits not classified and expenditures on research and development in the sectors in question. Due to missing data, in three countries, Germany, Poland, and Slovakia, social benefits other than social transfers in kind are extended with social transfers in-kind – purchased market production. Its effect is marginal in education and social protection expenditures, but it likely creates a minor overlap of cash and in-kind spending on health care. The use of final consumption expenditures, instead of individual consumption expenditures, is also made necessary by missing data in Denmark, Germany, and the United Kingdom. In education, health, and social protection, final and individual consumption expenditures fully overlap except for expenses on research and development (R&D) and items not elsewhere classified (n.e.c.). Since both R&D and n.e.c. items are age- and SES-neutral pure public goods (‘collective consumption expenditures’), the final and the individual consumption expenditures are interchangeable for the purposes of our calculation.

### Taxes

On the taxation side, calculations concerning the revenues of the welfare system start with the assumption that benefits and taxes balance each other: the system raises as much as it spends. What is not covered by earmarked taxes, such as social contributions, is financed from what we call general taxation, which is a composite of government revenue other than social contributions. Without this assumption, calculating net benefits would be meaningless.

The age- and SES-distribution of taxes financing the welfare state differs from the age- and SES-distribution of all taxes. This points to an asymmetry between the expenditure and the revenue sides. While, as mentioned above, there is practically no distribution by age and SES beyond the welfare state on the expenditure side, the finances of pure public goods are redistributive both by age and SES. The asymmetry does not affect our conclusions since our analysis is limited to the welfare state.

*Direct taxes*

Taxes levied on labor income (including payroll tax) are reported in EU-SILC at the household level. Such direct taxes are divided between household members by their labor income.

*Indirect taxes*

Payment of indirect taxes (VAT and excise duty) is estimated using data from the HBS, which contains detailed information on the household consumption of various goods and services. Individual VAT payments are calculated from individual consumption expenditures and VAT rates. Average VAT rates by main COICOP categories were taken from (9). Individual consumption expenditure is derived from household aggregates using the OECD II equivalence scale. Excise duty is levied on the consumption of tobacco, alcohol, and fuel. The latter was estimated from HBS data using weights developed by experts at the Hungarian Central Statistical Office to split consumption among household members. As the HBS only provides information on spending on cigarettes and alcohol (and not on the quantities consumed), we used information on quantities consumed from EHIS data.

To keep our set of incidence assumptions consistent, we assigned taxes levied on children’s consumption first to the children; we assigned them to the parents in the robustness test.

Direct and indirect taxes are estimated from various sources. To enable us to handle them simultaneously, we imputed VAT and excise duty payments to EU-SILC. As in other studies that analyze the redistributive effect of indirect taxes – such as (3) or (4) – we used a regression-based method for the imputation. (10) present a range of common variables in HBS and EU-SILC. For the majority of these variables, the distributions are highly comparable between the two datasets. We use the following variables to predict household VAT payment: gender of household head, age of household head, percentage of household members below age 5, percentage of household members between age 6 and 14, percentage of household members aged 70 years or older, urbanization (densely populated, intermediate, thinly populated), region, household size, household type (six categories), highest education level of household head (less than upper secondary, upper secondary, tertiary), economic activity of household head (employed, unemployed, retired, inactive), occupation of household head (10 categories) and log household income. We constructed a model of VAT payments in the HBS, based on overlapping socio-demographic variables as explanatory variables, and applied this model to predict the VAT payment of households in the EU-SILC. A similar method was used to impute alcohol and tobacco consumption from EHIS into EU-SILC in estimating the age and SES profiles of excise duties.

## Macro adjustment of the tax profile

Finally, all tax items assigned to taxpayers were readjusted to the aggregates reported in the Excise Duty Tables of the Directorate-General for Taxation and Customs Union of the European Commission and the National Tax Lists tables of Eurostat.

## Measurement of socio-economic status

SES is construed at the household level, assuming that household members have the same status. Public cash benefits are part of the dependent variable. They constitute a substantial component of income especially in old age. Applying a non-income measure of SES protects our regressions from endogeneity.

We constructed a composite indicator that measures material living standards on an interval scale; enables the formation of equal-size groups; but is not based on income. SES scores were created by combining information on the education and occupation of household members, as well as information on material living standards and housing.

Education was measured by the number of years spent in full-time education. The original EU-SILC variable (which measures the highest level of education attained) has been converted into years of education, based on Table A1.1 in (5). Education at the household level was defined as the average number of years spent at school by adult household members older than 21.

As for occupation, the EU-SILC records actual occupation (ISCO-88) for the current main job (for those of active age) or the last main job (for the inactive). These codes were converted to International Socio-Economic Index of Occupational Status (ISEI) scores using (6) methods. Again, occupation at the household level was measured by the mean score of adult household members.

The measure of material deprivation is based on the nine items of EU-SILC that assess the financial stress facing a household and the durables it can afford. These are the ability to absorb unexpected expenses; to have a week-long holiday away from home; to afford a meal containing meat or fish every second day; to keep the house adequately warm; to have no arrears on mortgage, rent, utility bills, or other loans; to have a washing machine; to have a color TV; to have a telephone; and to have a car. These items are the same as those used by Eurostat to construct the severe material deprivation indicators (see (7)). However, instead of adopting a cut-off, we add up the 0–1 indicators to arrive at a material deprivation score.

Our housing scores are based on the Eurostat measures of household deprivation, such as having a leaking roof, damp walls, floors, or foundations; not having a bath or shower in the dwelling; not having an indoor flushable toilet for the sole use of the household; and the dwelling being too dark. These 0–1 indicators are added up to form an indicator of housing deprivation. A further variable measuring overcrowdedness is added, based on the number of rooms relative to the number of inhabitants. Again, we followed the definition of (8).

The composite indicator of a household’s economic status is constructed from the basic dimensions of education, occupation, material deprivation, and housing by principal component analysis.

## References

1. United Nations. National Transfer Accounts Manual. New York: UN; 2013.

2. Verbist G, Förster M. F, Vaalavuo M. The impact of publicly provided services on the distribution of resources: Review of new results and methods. Paris; 2012. (OECD Social, Employment and Migration Working Papers). Report No.: 130.

3. De Agostini P, Capeau B, Decoster A, Figari F, Kneeshaw J, Leventi C, et al. EUROMOD Extension to Indirect Taxation: Final Report. Vol. 2017, Euromod Technical Note. Colchester; 2017.

4. Pestel N, Sommer E. Shifting Taxes from Labor to Consumption: More Employment and more Inequality? Rev Income Wealth. 2017;63(3):542–63.

5. OECD. PISA 2012 Results: Excellence Through Equity: Giving Every Student the Chance to Succeed. Vol. II. Paris; 2013.

6. Ganzeboom HBG, Treiman DJ. International Stratification and Mobility File: Conversion Tools [Internet]. 2010. Available from: http://www.harryganzeboom.nl/ismf/index.htm

7. Guio A-C. What can be learned from material deprivation indicators in Belgium and in its regions? Luxembourg; 2009. (Eurostat Methodologies and Working Papers).

8. Eurostat. Statistics explained. Housing statistics. [Internet]. 2017 [cited 2018 Jun 12]. Available from: http://ec.europa.eu/eurostat/statistics-explained/index.php/Housing_statistics

9. CPB. A study on the economic effects of the current VAT rates structure. Addendum: Country analyses. The Hague; 2013.

10. Serafino P, Tonkin R. Statistical matching of European Union statistics on income and living conditions (EU-SILC) and the household budget survey [Internet]. Luxembourg; 2017. (Eurostat Statistical Working Papers). Available from: http://ec.europa.eu/eurostat/web/income-and-living-conditions/publications/-/asset_publisher/zdEOYZhr9af3/content/KS-TC-16-026/3888793?inheritRedirect=false
