# Supplementary material for: Welfare states as lifecycle redistribution machines: Decomposing the roles of age and socio-economic status shows that European tax-and-benefit systems primarily redistribute across age groups
Source: PLoS One. 2021 Aug 25;16(8):e0255760. doi: 10.1371/journal.pone.0255760 (PMC8386825; doi:10.1371/journal.pone.0255760)
Supplement: S2 File — (DOCX) [file pone.0255760.s010.docx]

**S4 File. Models with control variables and country dummies.**

We checked the robustness of the effects of age and SES by adding control variables, such as gender, household size, migration status, and degree of urbanization to the model. Household size is introduced as a continuous variable, not as a group of dummies, so the coefficient-based statistics (standard deviation, sum of absolute values) are not applicable. Migration status has three categories: non-migrant (the reference category), migrant from another EU-country, and migrant from beyond the EU. Urbanization also has three categories: densely populated (the reference category), intermediate, and thinly populated. We also added country dummies to the model, but no interaction terms are included.

S4 Table presents similar summary statistics of the standardized regression coefficients as above: the standard deviation and the mean of the coefficients’ absolute values. Instead of the sum, we use the mean of the absolute values because the variable-groups do not consist of the same number of variables. Besides, we present the results of the Shapley-value decomposition of the relative contributions to the explained variance.

**S4 Table. Standard deviation and mean absolute value of regression coefficients and relative contribution to the explained variance for benefits, taxes, and net benefits by age, SES, and control variables.**

|  | Standard deviation | | | Mean absolute values | | | Relative contribution to *R^2^* (%) | | |
| --- | --- | --- | --- | --- | --- | --- | --- | --- | --- |
|  | Benefits | Taxes | Net benefits | Benefits | Taxes | Net benefits | Benefits | Taxes | Net benefits |
| Age | 0.15 | 0.11 | 0.14 | 0.12 | 0.17 | 0.15 | 83 | 51 | 80 |
| SES | 0.01 | 0.08 | 0.06 | 0.02 | 0.09 | 0.07 | 1 | 33 | 17 |
| Gender | 0.04 | 0.07 | 0.03 | 0.03 | 0.05 | 0.02 | 1 | 4 | 1 |
| Household size | - | - | - | - | - | - | 7 | 2 | 0 |
| Migration status | 0.01 | 0.01 | 0.01 | 0.01 | 0.01 | 0.00 | 0 | 1 | 0 |
| Urbanization | 0.01 | 0.02 | 0.00 | 0.01 | 0.02 | 0.01 | 0 | 2 | 1 |
| Country | 0.03 | 0.03 | 0.01 | 0.02 | 0.02 | 0.01 | 8 | 6 | 1 |

Notes: Absolute contributions sum to model R^2^, while relative contributions sum to 100%. Household size is added as a continuous variable, not as a group of dummies, so the standard deviation and the mean absolute values are not applicable.

The results of the article prove to be robust. Age dominates the benefits model, and SES remains marginal. If any of our previous conclusions has to be modified, it is about the tax model. When gender and household size are included in the model, SES is even less on par with age. The latter is clearly more important in all three ways of measurement. It is also evident that none of the control variables is nearly as important as age.
